# Load-sharing biomechanics of lumbar fixation and fusion with pedicle subtraction osteotomy

**DOI:** 10.1038/s41598-021-83251-8

**Published:** 2021-02-11

**Authors:** Luigi La Barbera, Hans-Joachim Wilke, Maria Luisa Ruspi, Marco Palanca, Christian Liebsch, Andrea Luca, Marco Brayda-Bruno, Fabio Galbusera, Luca Cristofolini

**Affiliations:** 1 Laboratory of Biological Structure Mechanics, Department of Chemistry, Materials and Chemical Engineering “G. Natta”, Politecnico Di Milano, Piazza Leonardo da Vinci 32, 20133 Milan, Italy; 2 Institute of Orthopaedic Research and Biomechanics, Trauma Research Center Ulm, Ulm University, Ulm, Germany; 3 Department of Industrial Engineering, School of Engineering and Architecture, Alma Mater Studiorum, Università Di Bologna, Bologna, Italy; 4 Department of Spine Surgery III, IRCCS Istituto Ortopedico Galeazzi, Milan, Italy; 5 IRCCS Istituto Ortopedico Galeazzi, Milan, Italy

**Keywords:** Biomedical engineering, Experimental models of disease, Translational research, Bone quality and biomechanics

## Abstract

Pedicle subtraction osteotomy (PSO) is an invasive surgical technique allowing the restoration of a well-balanced sagittal profile, however, the risks of pseudarthrosis and instrumentation breakage are still high. Literature studied primary stability and posterior instrumentation loads, neglecting the load shared by the anterior column, which is fundamental to promote fusion early after surgery. The study aimed at quantifying the load-sharing occurring after PSO procedure across the ventral spinal structures and the posterior instrumentation, as affected by simple bilateral fixation alone, with interbody cages adjacent to PSO level and supplementary accessory rods. Lumbar spine segments were loaded in vitro under flexion–extension, lateral bending, and torsion using an established spine tester. Digital image correlation (DIC) and strain-gauge (SG) analyses measured, respectively, the full-field strain distribution on the ventral surface of the spine and the local strain on posterior primary rods. Ventral strains considerably decreased following PSO and instrumentation, confirming the effectiveness of posterior load-sharing. Supplemental accessory rods considerably reduced the posterior rod strains only with interbody cages, but the ventral strains were unaffected: this indicates that the load transfer across the osteotomy could be promoted, thus explaining the higher fusion rate with decreased rod fracture risk reported in clinical literature.

## Introduction

Pedicle subtraction osteotomy (PSO) is a challenging surgical technique performed through a posterior approach, resecting major parts of the vertebral body (VB), pedicles, and bony structures to restore a well-balanced sagittal profile^1^. Posterior fixation with pedicle screws and spinal rods is always required to stabilize the spine and to achieve long-term fusion. PSO is also accompanied by a high rate of post-operative complications mainly related to rod fracture (16–39%) and malunion or pseudarthrosis (12–31%) at the treated level^2–9^.

The recent literature contributed in better understanding of the basic biomechanics behind PSO fixation. Clinical studies reported lower pseudarthrosis and implant failure rates with multi-rod constructs and interbody cages implantation^2–5,10^. Previous in vitro studies demonstrated the effectiveness of accessory and satellite rods supplemented with adjacent cages in providing adequate motion restriction or primary stability^11–17^, while reducing the strains on the spinal rods^11–13^. Computational studies based on finite element analysis also reported similar findings^18,19^.

The past literature did not address how the load are transferred through the anterior column. Well-established intradiscal pressure (IDP) measurements have often been used as an index of the loads transferred through the anterior column of intact and instrumented spine segments, following a destabilization of one or more functional spine units (FSUs) due to damage of the soft tissues (e.g. nucleotomy, ligaments resections), bony structures (e.g. VB fracture) or both (e.g. laminectomy)^20–22^, however neglecting the biomechanical contribution of the remaining bony structures and ligaments. Other studies relied on strain gauges (SG) to investigate how the cortical shell of the vertebra is shielded by posterior instrumentation^23^, thus neglecting the surrounding soft tissues and providing only pointwise measurement on bone. The load transfer between the column and the posterior fixation is of great interest in the setting of PSO fixation and fusion, where the integrity of the anterior longitudinal ligament (ALL) and the anterior “bony bridge” is preserved to intraoperatively guide osteotomy closure in order to post-operatively stabilize the anterior spine and to promote fusion early after surgery, but the current measurements are not able to fully appreciate this process. In this context, an experimental approach based on Digital Image Correlation (DIC) could offer the potential to measure the superficial full-field strain distribution on hard and soft-tissue, both on the treated vertebral body (VB), the adjacent intervertebral disc (IVD), as well as the ligamentous structures. The potential of such an approach has been recently demonstrated for intact porcine^24,25^ and human^26,27^ poly-segmental spine specimens.

No prior study ever investigated the strain distribution on the anterior column of an instrumented spine following PSO, nor how the strain is related to the usage of posterior fixation and anterior interbody fusion. We hypothesized that the load-sharing mechanism, well-accepted for an instrumented spine segment^28^, may play a decisive role also in the setting of PSO instrumentation: the loads on the anterior column may promote bone remodelling and fusion of the osteotomy rims, while the loads shielded by the posterior instrumentation may be responsible of posterior rod breakage due to cyclic loads. Therefore, the aim of the current study was to quantify how the load supported by the ventral spinal structures and the posterior instrumentation is affected both by the number of rods and the implantation of anterior supports adjacent to PSO. In particular, the present study focused on the variations in biomechanics due to alternative instrumentation techniques (simple bilateral fixation, additional interbody cage implantation, and usage of supplementary accessory rods) following PSO through in vitro flexibility tests integrated with DIC strain measurement on the ventral spine and using strain gauge analysis on the posterior primary rods.

## Material and methods

### Specimens

Three fresh-frozen human spine specimens were collected via an ethically approved donation program (Science Care Inc., Phoenix, AZ). The study was approved by the Institutional Review Board (Ethikkommission) of Ulm University (Document of approval Nr. 307/17).

All specimens underwent sagittal X-rays (Faxitron 43,805 N, Hewlett Packard, Palo Alto, USA) and clinical CT-scans (Philips Brilliance 64, Philips Healthcare, Cleveland, USA) to assess the bone mineral density (BMD) and to exclude any defect, tumour or severe degeneration (Table 1). Specimens, stored at − 20 °C, were thawed at 6 °C for about 10 h prior to preparation, including muscle and fat tissue removal, and testing, both performed within 20 h to avoid degradation in terms of mechanical response. Half of the cranial and the caudal vertebrae were embedded in bone cement (Technovit 3040, Heraeus Kulzer, Werheim, Germany).

**Table 1:** Specimen data.

Specimen	Segment	Sex	Age at death (years)	Height (cm)	Weight (kg)	BMD (mg/cm^3^)	Lumbar lordosis (°)	Intervertebral disc degeneration^29^
#1	T11-S1	M	66	183	141	82.5 ± 2.3	39	Grade 1 (mild degeneration)
#2	T11-S1	M	62	178	164	94.4 ± 3.6	39	Grade 2 (moderate degeneration)
#3	T11-S1	F	60	163	114	122.6 ± 7.4	38	Grade 2 (moderate degeneration)

### Surgical constructs

**Figure 1:**
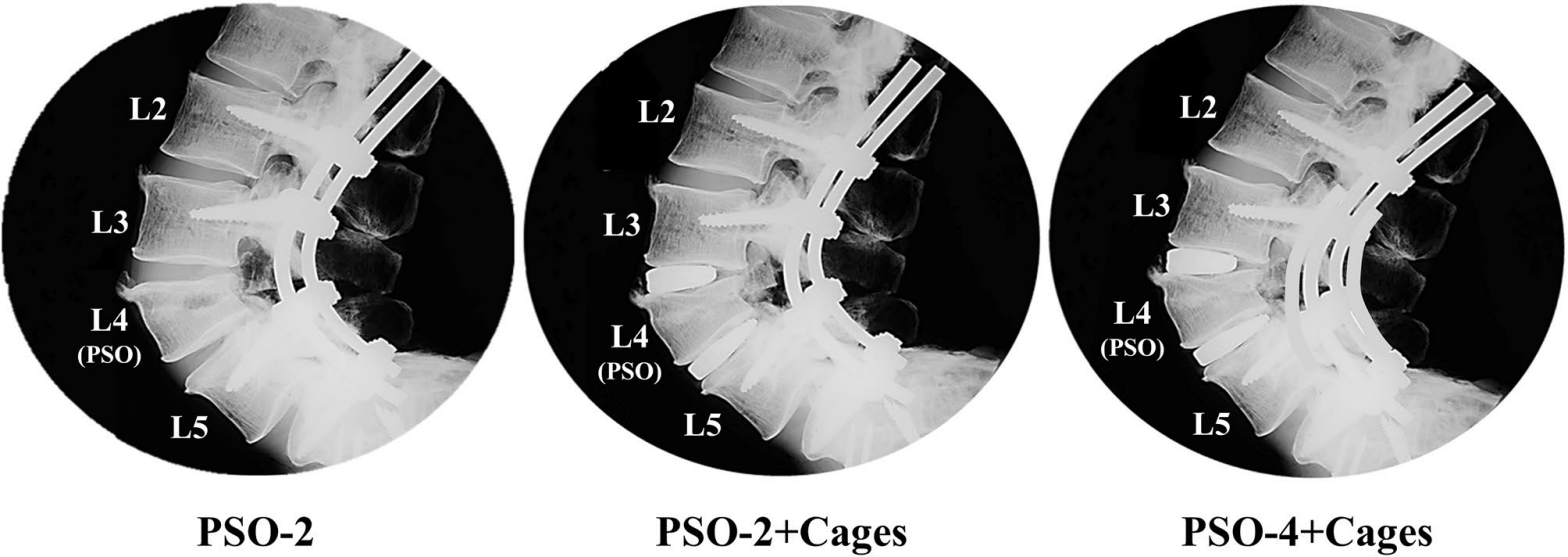
Sagittal X-ray scans of a representative specimen reporting the three steps of instrumentation.

The intact specimens underwent the following three steps of instrumentation (Fig. 1):

- “PSO-2”: the PSO (Fig. 1) was performed at L4, setting a target lordosis of 60°, followed by bilateral fixation with pedicle screws (Expedium polyaxial screw system 5.5–6.5 × 40–45 mm, DePuy Synthes, Raynham, MA, USA; CD Horizon Legacy 5.5–6.5 × 40-45 mm, Medtronic Sofamor Danek, Minneapolis, MN, USA) and 5.5 mm CoCr spinal rods (Medtronic, Minneapolis, MN, USA) from L2 to S1;
- “PSO-2 + Cages”: two 10° XLIF cages (8/10 × 22x55 mm, NuVasive, San Diego, CA) were introduced adjacent to PSO level following lateral excision of L3-L4 and L4-L5 IVDs. The accurate preparation of the disc space, which was best fitted with the appropriate cage size ensured a wide footprint on the outer margins of the endplates;
- “PSO-4 + Cages”: the construct was instrumented with a pair of supplementary accessory rods fixed with dominos (Medtronic, Minneapolis, MN, USA) to the primary ones.

The two experienced surgeons participating in the study (MBB, AL) took care of specimens’ preparation and implants’ selection according to their routine surgical practice. Each instrumentation step was followed by a biomechanical test as described below.

### Flexibility tests

Quasi-static loading was applied using a well-established spine tester^30^, equipped with three stepper motors (Isel 3450, Isert-electronic, Eiterfeld, Germany) and a 6-DOF load cell (FT1500/40, Schunk GmbH &Co.KG, Lauffen/Neckar, Germany). The caudal end of the specimen was fixed to the testing apparatus, while the cranial end was loaded for 3.5 cycles under pure bending moments up to ± 7.5 Nm in flexion/extension (FE), right/left lateral bending (LB), and right/left (or clockwise/counter-clockwise) axial torsion (AT), at a constant angular velocity (1.0°/s in FE and LB, 0.5°/s in AT). To avoid viscoelastic effects, the third loading cycle served for data analysis^31^.

For motion analysis, three reflective markers were screwed on each vertebra. The kinematics of each vertebra was captured using a motion tracking device based on six Vicon MX13 cameras (Vicon Motion Systems Ltd., Oxford, UK). The error on angle measurement was < 0.1°^32^. The kinematics of each functional spine unit was matched with the moment data to determine the resulting moment–angle curve and to calculate the local (L3–L5) range of motion (RoM) and neutral zone (NZ) using a script in MATLAB R2014b (MathWorks, Natick, MA, USA).

### DIC on the ventral spine

To measure the full-field strain distribution with the DIC on the ventral aspect of the spine, flexibility tests were repeated. The DIC system (Q400, Dantec Dynamics, Denmark) based on two 5 Mpixel cameras (2440 × 2050, 8-bit, black-and-white) with high-quality metrology standard 17 mm lens (Xenoplan, Schneider-Kreuznach, Germany; 35 mm equivalent focal length of 65 mm), allowing to acquire images of the specimens through a stereoscopic vision.

The specimens were placed at a distance of 540 mm from the cameras, which were vertically aligned to include L4 vertebra and the adjacent IVDs (L3–L4 and L4–L5) within the region of interest (ROI). In this configuration, the field of view was of about 120–160 mm (depending on the specific specimen), resulting in a pixel size of about 0.08 mm, and a depth of field of 70 mm with the aperture adopted (f/22). ROI images were acquired at five frames per second. Lighting of the specimen was carried out with a directional custom system of LEDs (10,000 lm in total).

To allow stereoscopic reconstruction and to minimize the distortion of the lenses, a calibration was performed before each test using a calibration target (Al4-BMB-9 × 9, Dantec Dynamics). Proprietary software Istra 4D (v4.3.1, Dantec Dynamics, Denmark) was used to measure the 3D displacement field and to calculate the strains. The maximum (ε_1_) and minimum (ε_2_) engineering principal strains and their direction were computed keeping consistent software parameters (facet size: 39–59 pixels; grid spacing: 4 pixels; contour smoothing: kernel size 5 × 5), obtained by former validation and optimization^27,33,34^.

To estimate the measurement uncertainties, a couple of images for each specimen in the unloaded condition were taken before each test. The systematic and random errors were evaluated in terms of mean and standard deviation of the components of strain over the entire ROI.

The full-field strains were computed by the DIC during all cycles, considering only the third one for data analysis^31^. For a qualitative analysis, the strain maps, from L4 VB to L4-L5 IVD, were reported for each loading mode (F/E, right/left LB, right/left AT) and for each surgery step (Intact, “PSO-2”, “PSO-2 + Cages”, “PSO-4 + Cages”) at maximum and minimum load.

**Figure 2:**
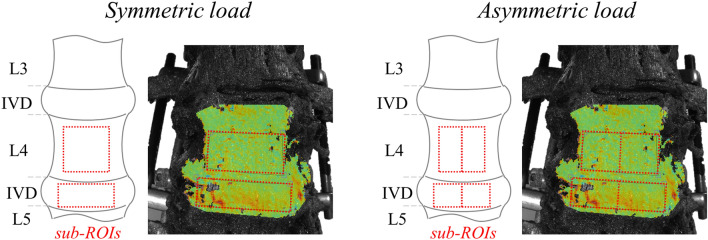
Identification of sub-regions of interest (sub-ROIs) used for median strain values extraction on a representative specimen (Specimen #2, anterior view) and schematic drawing of corresponding spinal structures. One unique sub-ROI was used in flexion–extension (left) in correspondence of the L4 VB (PSO level) and another of the L4-L5 IVD, while two sub-ROIs were used in lateral bending and torsion (right). The DIC strain maps have been obtained using the proprietary software Istra 4D (v4.3.1, Dantec Dynamics, Denmark; URL: https://www.dantecdynamics.com/).

Quantitative analysis was performed at full load using a MATLAB script (R2019b, MathWorks, Natick, USA), extracting the median strain value on specific sub-regions of interest (sub-ROIs, Fig. 2), then normalized with respect to “Intact” condition. Being the load symmetric in flexion–extension on both sides of the specimen, one sub-ROI in front of L4 vertebra and another on L4–L5 IVD were considered, resulting in 3 median strain values (one per specimen) for each loading condition. Being the load asymmetric in lateral bending and axial torsion, to discriminate the difference in strain distribution arising on each side of the specimen, the sub-ROIs were furtherly divided in two symmetrical regions, resulting in 6 median strain values for each loading condition.

### Strain gauge analysis on primary rods

During the flexibility tests, the primary rods at PSO level were posteriorly instrumented with rectangular strain gauge (SGs) rosettes (KFG-2-120-D17-11L1M2S; Kyowa Electronic Instruments Co. Ltd, Tokyo, Japan) on the most posterior part of the rods^11,12,35,36^. Compensation of thermal effects was achieved connecting each SG to a dummy compensator in a half-bridge configuration to a MX840B (HBM, Darmstadt, Germany) amplifier. Strain signals were zeroed before testing and then sampled at 10 Hz. The strains on each rosette were combined to calculate the maximum (tensile) and minimum (compressive) principal strains on all loading cycles in every motion direction for each rod, considering only the third one for data analysis. Primary rod strains collected on both SG rosettes were considered as independent, resulting in 2 values per specimen for each loading condition either at maximum and minimum load. Data were normalized to “PSO-2” condition. Only tensile values, considered to be more critical, will be discussed herein.

### Data analysis and statistics

Statistical differences on strain variance due to each instrumentation step (“PSO-2”, “PSO-2 + Cages”, “PSO-4 + Cages”), were calculated in Statistica (v13, TIBCO software Inc, Tulsa, OK) using a non-parametric Kruskal–Wallis test and Dunn post-hoc correction of the significance level p* = 2p/(K(K − 1), with K = number of multiple comparisons and p = 0.05.

To discriminate asymmetric loading conditions, strain data were grouped as “ipsilateral”, with the load directed on the same side where the sub-ROI and the SG rosette were located (e.g. left sub-ROIs and left primary rod are ipsilateral in left LB), and “contralateral”, with the load directed on the opposite side compared to where the sub-ROI and the SG rosette were located (e.g. right sub-ROIs and right primary rod are contralateral in left AT).

## Results

### Flexibility tests

The RoM was consistent for all intact specimens with higher values in flexion–extension and lateral bending compared to torsion (Table 2, Supplementary Table 1). Following PSO and bilateral instrumentation (“PSO-2”), the RoM approached the error of measurement (0.1°) without relevant differences compared to cages implantation (“PSO-2 + Cages”) and addition of supplementary rods (“PSO-4 + Cages”) both in flexion/extension and lateral bending (below 4% of the intact condition), while torsional values were relatively higher (about 20% of intact). The neutral zone (NZ) followed the same qualitative trend of RoM, with lower initial values (Table 2, Supplementary Table 1).

**Table 2:** Median [min; max] local (L3–L5) range of motion (RoM) and neutral zone (NZ) values for each instrumentation step.

	Flexion/extension		Lateral Bending		Axial Torsion	
	RoM (°)	NZ (°)	RoM (°)	NZ (°)	RoM (°)	NZ (°)
Intact	12.0 [9.7; 14.5]	3.7 [1.9; 5.5]	12.8 [12.4; 13.6]	4.4 [3.7; 5.3]	7.8 [5.4; 8.5]	0.8 [0.8; 1.8]
PSO-2	0.5 [0.0; 0.6]	0.0 [− 0.1; 0.1]	− 0.1 [− 0.3; 0.0]	0.0 [0.0; 0.0]	1.5 [1.4; 1.7]	0.1 [0.1; 0.1]
PSO-2 + Cages	0.3 [− 0.5; 0.3]	0.1 [0.1; 0.1]	0.0 [0.0; 0.0]	0.0 [0.0; 0.1]	1.5 [1.5; 1.5]	0.2 [0.0; 0.2]
PSO-4 + Cages	0.2 [− 0.4; 0.4]	− 0.1 [0.0; − 0.1]	0.0 [0.0; 0.0]	0.0 [0.0; 0.1]	1.4 [1.2; 1.4]	0.1 [0.1; 0.2]

Data for each specimen provided as Supplementary Table 1.

### General trends—Strain distribution on the ventral spine and on primary rods

The systematic and random errors over the entire ROI were, respectively, lower than 20 and 60 µstrains.

**Figure 3:**
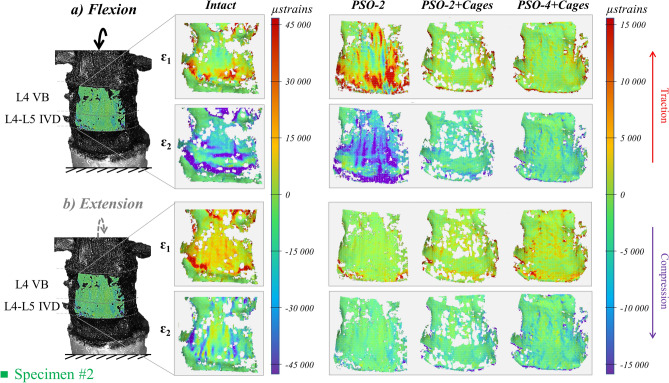
Flexion (**a**) and extension (**b**): tensile (ε_1_) and compressive (ε_2_) strain maps measured on the “Intact” condition and following PSO at L4 and posterior instrumentation with 2 primary-rods (“PSO-2”), with 2 rods and supplementary intervertebral cages (“PSO-2 + Cages”), and with supplementary accessory rods and intervertebral cages (“PSO-4 + Cages”). A picture of the specimen with the correlated areas are reported on the left, indicating the treated level (L4) and the caudal IVD. A representative specimen (#2) is shown here (strain maps for every specimen provided as Supplementary Figures). The DIC strain maps have been obtained using the proprietary software Istra 4D (v4.3.1, Dantec Dynamics, Denmark; URL: https://www.dantecdynamics.com/).

The spine segments demonstrated highly inhomogeneous ventral strain distribution in the intact condition, with lower values on L4 than on the L4-L5 IVD (Figs. 3, 5, 7, strain maps of every specimen provided as Supplementary Figures, strain data provided as Supplementary Table 2). Following PSO, a general decrease in ventral strains was noticed compared to the intact condition in all loading directions, while lower changes were observed among PSO configurations.

#### Flexion

**Figure 4:**
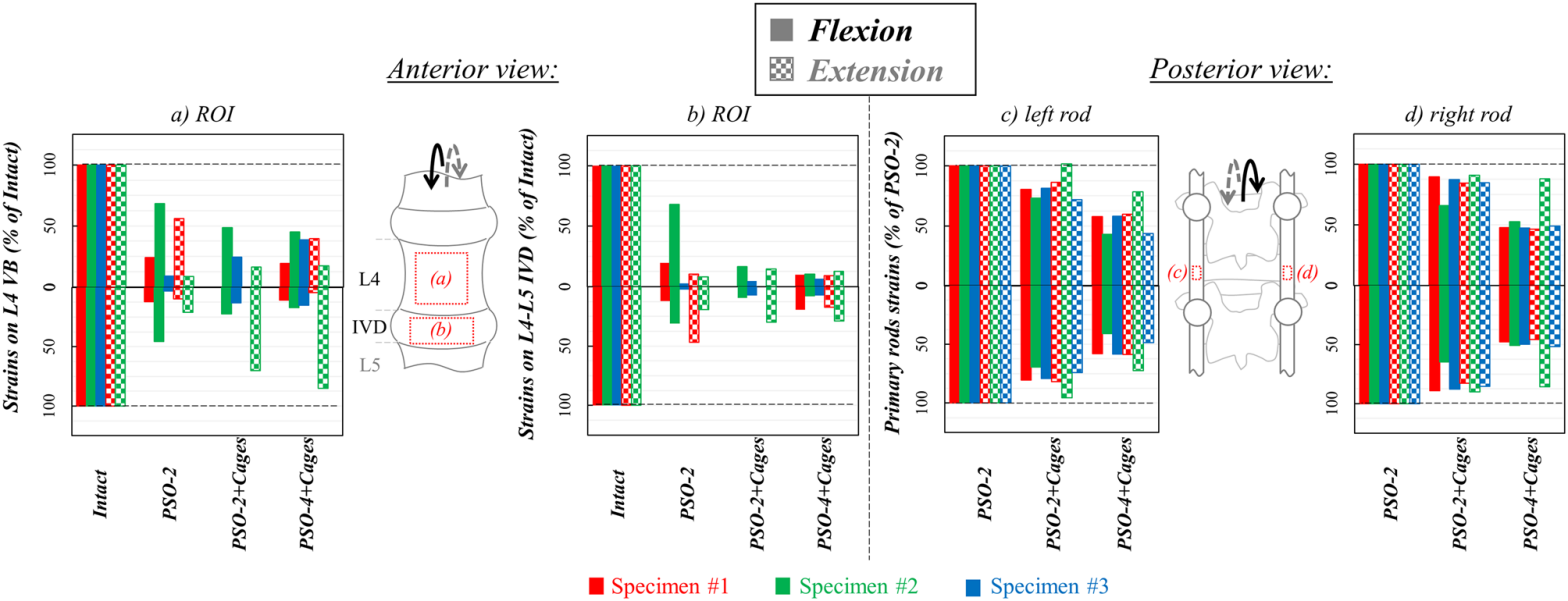
Flexion/extension: Normalized strain values on the regions of interest (ROI) on L4 VB (**a**) and on the L4–L5 IVD (**b**) and normalized primary rods strains both on the left (**c**) and right (**d**) rods following PSO at L4 and posterior instrumentation with 2 primary-rods (“PSO-2”), with 2 rods and supplementary intervertebral cages (“PSO-2 + Cages”) and with supplementary accessory rods and intervertebral cages (“PSO-4 + Cages”). Supplementary Tables provide quantitative strain data either for each specimen both anteriorly and posteriorly.

In the intact condition, compressive strains were directed in axial direction (Fig. 3a) and they were higher on the L4-L5 IVD (− 23,000 µstrains), while being lower on the L4 VB (− 15,300 µstrains); tensile strains were directed transversally and they were comparable on the IVD (18,600 µstrains), but much lower on the VB (4400 µstrains). Following PSO, median tensile and compressive strains significantly decreased compared to the intact condition (p = 0.014), respectively, to 30% and 16% on the VB, and to 12% and 10% on the IVD (Fig. 4a,b). No significant differences were noticed among different instrumentation steps.

The posterior side of the primary rods underwent longitudinal tension, with median rod strains being the highest (400 µstrains) for simple bilateral stabilization. Strains normalized to “PSO-2” decreased to 81% after cage insertion and to 50% with supplemental accessory rods (Fig. 4c,d). Significant variations were found for “PSO-4 + Cages” compared to “PSO-2” (p = 0.004).

#### Extension

In the intact condition, tensile strains were directed axially (Figs. 3.b) on the L4-L5 IVD (21,000 µstrains), while they were lower on the L4 VB (11,800 µstrains); transverse compressive strains were comparable on the VB (− 10,500 µstrains), but much lower on the IVD (− 8300 µstrains). Following PSO, median tensile and compressive strains significantly decreased compared to the intact condition (p = 0.037), respectively, to 17% and 10% at PSO level, and to 9% and 17% on the IVD (Fig. 4a,b). No significant differences were noticed among different instrumentation steps.

The posterior side of the primary rods underwent longitudinal compression, with median strains being lower than in flexion (110 µstrains) for simple bilateral stabilization. Normalized strains decreased to 88% after cage insertion and to 60% with supplemental accessory rods (Fig. 4c,d). Significant variations were found for “PSO-4 + Cages” compared to “PSO-2” (p = 0.011).

#### Lateral bending

**Figure 5:**
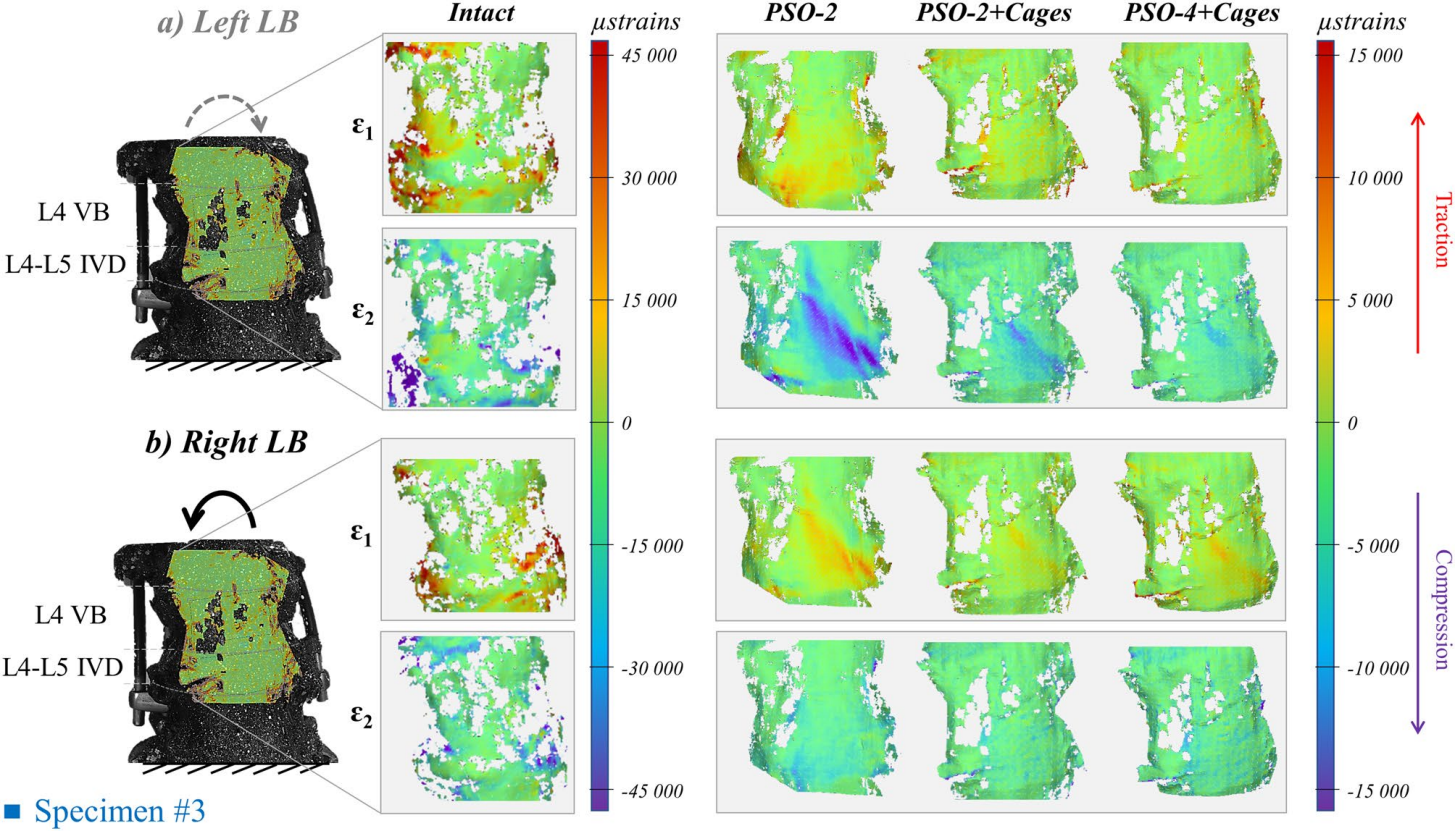
Lateral bending (LB) left (**a**) and right (**b**): tensile (ε_1_) and compressive (ε_2_) strain maps measured in the “Intact” condition and following PSO at L4 and posterior instrumentation with 2 primary-rods (“PSO-2”), with 2 rods and supplementary intervertebral cages (“PSO-2 + Cages”) and with supplementary accessory rods and intervertebral cages (“PSO-4 + Cages”). A picture of the specimen with the correlated areas is reported on the left, indicating the treated level (L4) and the caudal IVD. A representative specimen (#3) is shown here (strain maps for every specimen provided as Supplementary Figures). The DIC strain maps have been obtained using the proprietary software Istra 4D (v4.3.1, Dantec Dynamics, Denmark; URL: https://www.dantecdynamics.com/).

**Figure 6:**
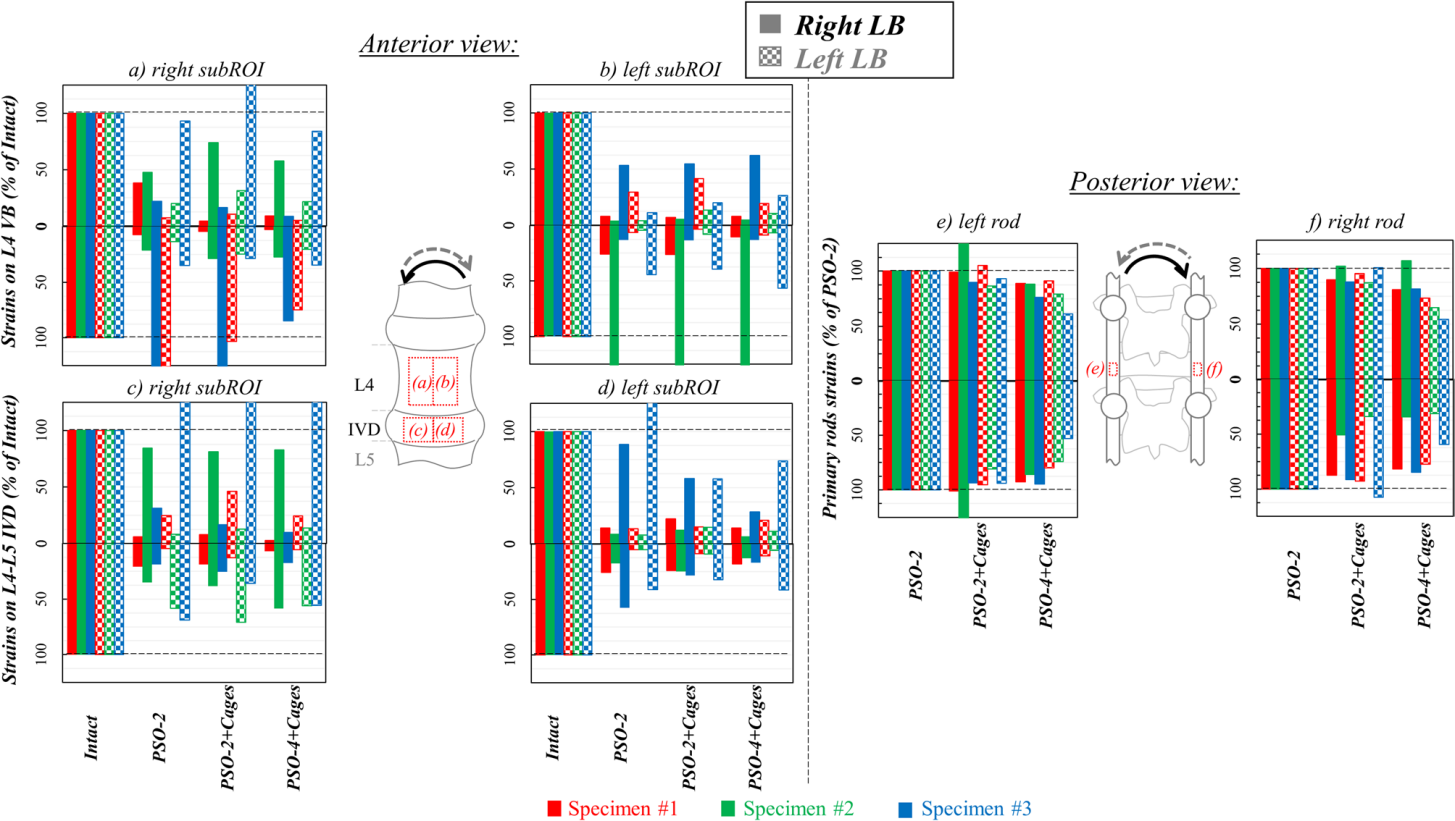
Lateral bending (LB): Normalized strain values on the sub-regions of interest (subROI) on L4 VB (**a** right subROI; **b** left subROI) and on the L4-L5 IVD (**c**: right subROI; **d** left subROI) and normalized primary rods strains both on the left (**e**) and right (**f**) rods following PSO at L4 and posterior instrumentation with 2 primary-rods (“PSO-2”), with 2 rods and supplementary intervertebral cages (“PSO-2 + Cages”) and with supplementary accessory rods and intervertebral cages (“PSO-4 + Cages”). Supplementary Tables provide quantitative strain data either for each specimen and grouped by ipsi-/contra-lateral sides both anteriorly and posteriorly.

In the intact condition, the median tensile and compressive strains were both comparable on the IVD (12,000 vs. − 11,000 µstrains) and higher than on the VB (5400 vs. − 7700 µstrains). Specimens underwent axial compression on the ipsilateral side with circumferential stretching on the IVD (Fig. 5), while the contralateral side underwent axial stretching and circumferential compression. Following PSO, ipsilateral tensile and compressive strains significantly decreased compared to the intact condition (p ≤ 0.002), respectively, to 18% and 13% at PSO level, and to about 17% and 15% on the IVD (Fig. 6a–d). On the contralateral side, tensile and compressive strains decreased to 14% and 29% at PSO level, and to about 17% and 20% on the IVD. Variations were significant in all cases (p ≤ 0.016), except on the contralateral compressive side of the VB. Significant variations were noticed on the ipsilateral compressive strains on the IVD for “PSO-2” and “PSO-4 + Cages” (p = 0.009) and on the contralateral tensile strains on the VB for “PSO-2” (p = 0.013). When grouping by number of rods (2 rods, 4 rods) and cage use, significant differences compared to intact were found (p ≤ 0.010).

The tensile and compressive rod strains were, respectively, higher on the ipsi- than the contralateral rod (185 vs. 66 µstrains) and lower on the contra- compared to the ipsi-lateral rod (− 230 vs- − 80 µstrains) in “PSO-2” configuration. Normalized tensile strains slightly decreased to 95% and 97%, respectively, on the ipsi- and contra-lateral rods after cage implantation and to 84% and 80% with supplemental accessory rods (Fig. 6e,f).

#### Axial torsion

**Figure 7:**
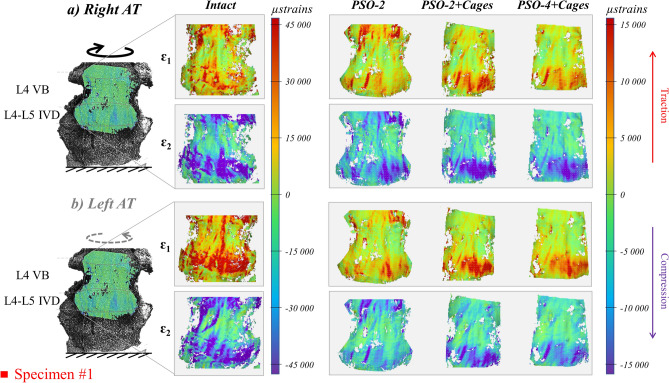
Axial torsion (AT) right (**a**) and left (**b**): tensile (ε_1_) and compressive (ε_2_) strain maps measured on the “Intact” condition and following PSO at L4 and posterior instrumentation with 2 primary-rods (“PSO-2”), with 2 rods and supplementary intervertebral cages (“PSO-2 + Cages”), and with supplementary accessory rods and intervertebral cages (“PSO-4 + Cages”). A picture of the specimen with the correlated areas is reported on the left, indicating the treated level (L4) and the caudal IVD. A representative specimen (#1) is shown here (strain maps for every specimen provided as Supplementary Figures). The DIC strain maps have been obtained using the proprietary software Istra 4D (v4.3.1, Dantec Dynamics, Denmark; URL: https://www.dantecdynamics.com/).

**Figure 8:**
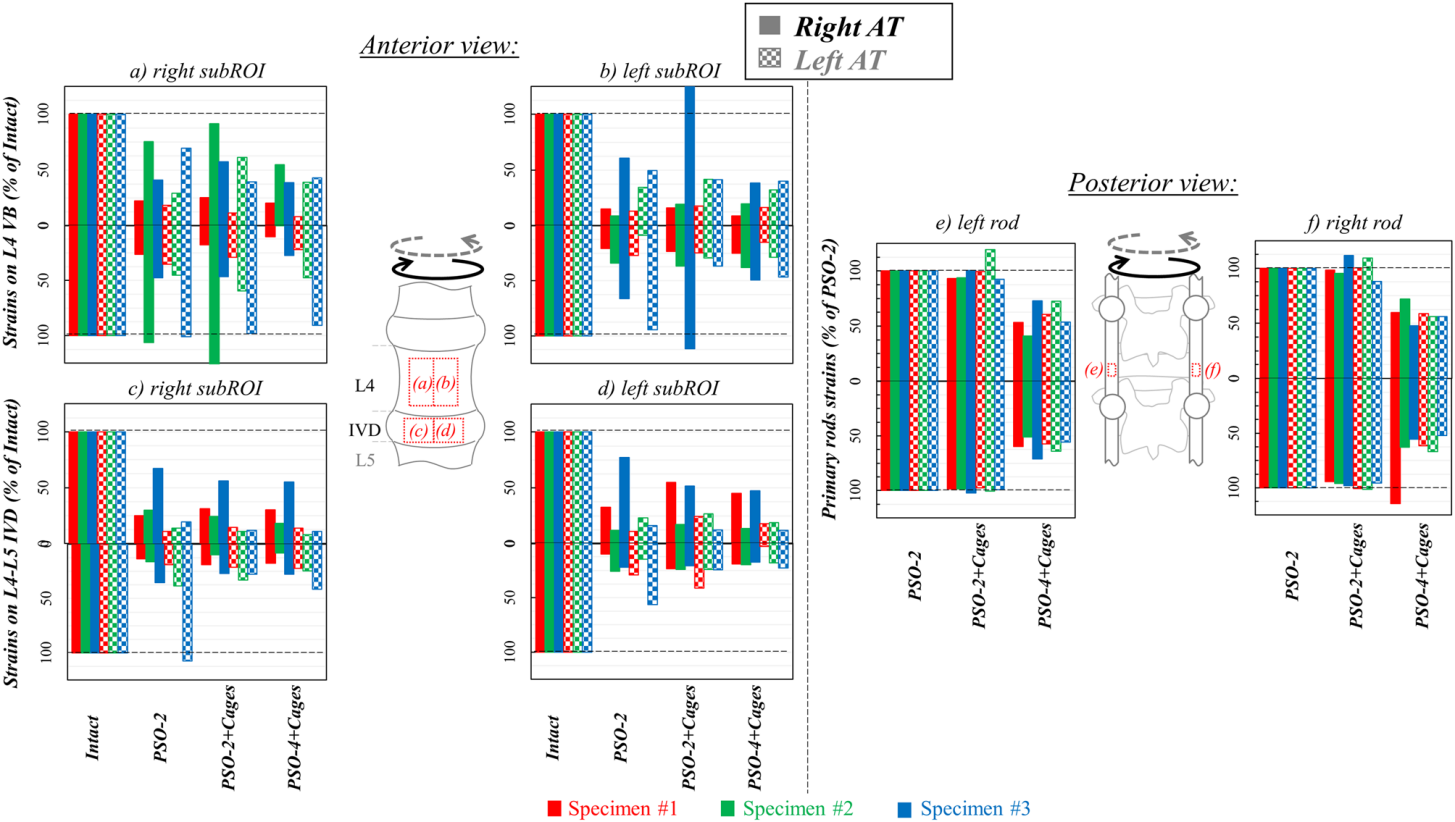
Axial torsion (AT): Normalized strain values on the sub-regions of interest (subROI) on L4 VB (**a**: right subROI; **b**: left subROI) and on the L4–L5 IVD (**c**: right subROI; **d**: left subROI) and normalized primary rods strains both on the left (**e**) and right (**f**) rods following PSO at L4 and posterior instrumentation with 2 primary-rods (“PSO-2”), with 2 rods and supplementary intervertebral cages (“PSO-2 + Cages”), and with supplementary accessory rods and intervertebral cages (“PSO-4 + Cages”). Supplementary Tables provide quantitative strain data either for each specimen and grouped by ipsi-/contra-lateral sides both anteriorly and posteriorly.

Torsion induced the highest tensile and compressive strains (about 27,300 and -30,300 µstrains, respectively) on the IVD of the intact specimens, while on the VB they were relatively lower (8300 and − 6600 µstrains). Tensile and compressive strains were oriented in the direction of load at about + 45° and − 45° (Fig. 7). Following PSO, ipsilateral tensile and compressive strains significantly decreased compared to the intact condition (p ≤ 0.011), respectively, to 35% and 31% at PSO level, to about 21% and 18% on the IVD (Fig. 8a–d); on the contralateral side, tensile and compressive strains significantly decreased to 34% and 49% at PSO level, to about 22% and 25% on the IVD (p ≤ 0.003). Significant variations were noticed for “PSO-4 + Cages” in the ipsilateral compressive strains on the IVD (p = 0.001) and in the contralateral tensile strains both at PSO level and on the IVD (p = 0.007).When grouping, significant differences compared to the intact condition were found when using 4 rods and cages (p ≤ 0.010).

The median tensile rod strains were slightly higher on ipsilateral rods, while the compressive strains were slightly higher on contralateral rods for simple bilateral stabilization. Normalized tensile strains kept rather constant both on the ipsi- and contra-lateral rods after cages implantation, but decreased, respectively, to 67% and 57% with supplemental accessory rods (Fig. 8e,f). Significant variations were found on the ipsilateral rod for “PSO-4 + Cages” compared to “PSO-2” (p = 0.015) and “PSO-2 + Cages” (p = 0.012).

### Specimen-specific analysis—strain distribution on the ventral spine and on primary rods

Each specimen demonstrated specific properties, both related to its size, BMD, lumbar lordosis before/after PSO procedure and the presence of mild to moderate degenerative signs (Table 1, Fig. 9). The strain maps of every specimen are provided as Supplementary Figs. 1–9, strain values as Supplementary Tables 2, 3.

**Figure 9:**
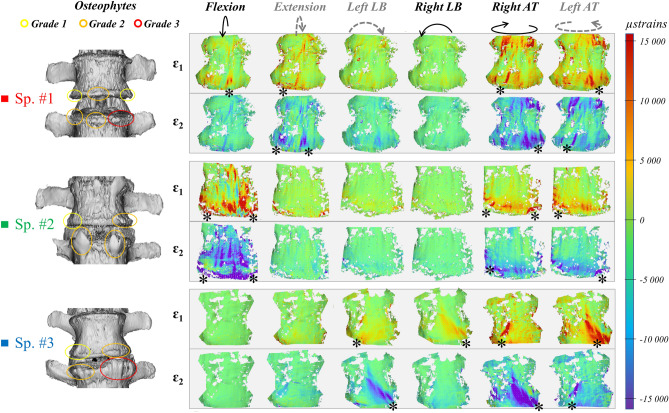
Specimen-specific analysis of maximum (ε_1_) and minimum (ε_2_) strain maps vs. osteophyte grade for cases “PSO-2” at full load for all loading conditions. CT reconstruction of each specimen (left) was used to grade osteophytes formations^26^. The asterisks (*) indicate the presence of strain intensification effects. The DIC strain maps have been obtained using the proprietary software Istra 4D (v4.3.1, Dantec Dynamics, Denmark; URL: https://www.dantecdynamics.com/).

#### Specimen #1

It exhibited mild degeneration (grade 1) with diffused osteophyte formations, which were more pronounced on the left inferior margin of L4-L5 IVD (Table 1, Fig. 9). Lumbar lordosis improved to 48° after PSO and posterior fixation. Although median strains were in line with the trends reported for other specimen, asymmetric loading was noticed particularly in torsion (Fig. 7), where the strains kept rather high even with cages and supplementary rods (Supplementary Figs. 1–3). Variability on normalized strains was rather high in specific cases across PSO configurations (Figs. 4, 6, 8). Following PSO, a general strain decrease was noticed on the IVD and at PSO level. IVD strains kept rather comparable in extension, lateral bending and torsion across different instrumentation steps. After cages insertion, a slightly decreasing trend on tensile strains was noticed in flexion both on the IVD (from 20% in “PSO-2” to 10% in “PSO-4 + Cages”) and on the VB (from 24 to 19%).

#### Specimen #2

It showed moderate degeneration (grade 2) with osteophytes on both sides of the IVD (Table 1, Fig. 9). Lumbar lordosis increased to 60° with PSO and posterior fixation. Strains higher than the median values reported for the other specimens were noticed in flexion/extension (Figs. 3, 4), with a symmetric map in torsion (Fig. 9, Supplementary Figs. 4–6). Normalized strains were higher than for other specimens in most loading conditions (Figs. 4, 6, 8). Normalized strains were comparable or slightly lower on the IVD than at PSO level in “PSO-2” configuration in flexion and torsion. A decreasing trend was noticed on the IVD following cages insertion (from 14–68% in “PSO-2” to 10% in “PSO-4 + Cages”), while normalized strains remained rather constant on the VB.

#### Specimen #3

It exhibited moderate degeneration (grade 2, Table 1, Fig. 9). Lumbar lordosis improved to 67° with PSO and posterior fixation. Osteophytes were more pronounced on the left inferior margin (grade 2–3) than on the right one (grade 1–2) of the IVD. High local asymmetric strain (even beyond ± 7800 µstrains) both in lateral bending and torsion were noticed (Figs. 5, 9, Supplementary Figs. 7–9), with relatively high variability on normalized strain values across PSO configurations (Figs. 4, 6, 8). Some similarities among the ventral strain maps in lateral bending and torsion were noticed (Fig. 9).

Following PSO, a consistent normalized strain decrease was noticed on the IVD in flexion and extension, with similar trends also at PSO level. Switching from “PSO-2” to “PSO-2 + Cages”, a relevant strain variation, more relevant for the IVD than for the VB, was noticed in compressive values in lateral bending and in torsion.

## Discussion

PSO is an invasive surgical technique allowing the restoration of a well-balanced profile in patients with severe sagittal imbalance^1^. PSO procedure is often accompanied by a high risk of rod breakage and pseudarthrosis at the osteotomy level due to the extensive resection through posterior, middle and anterior bony and ligamentous structures, featuring unique biomechanical challenges^2–9^.

Previous biomechanical studies demonstrated improved primary stability and reduced instrumentation loads using multi-rod constructs and additional interbody spacers^11–19^. However, they focused on the load supported by the posterior instrumentation in relation to rod breakage, neglecting the load supported by the anterior column, which is fundamental to promote fusion across the osteotomy early after surgery. While IDP measurements^20–22^ and SG techniques^23^ may offer a limited description on the actual loads on a specific structure, new DIC techniques^24–27^ can investigate the superficial full-field load (i.e. strain) distribution both on the VB treated with PSO and the adjacent IVDs, as well as the ALL running on the ventral spine. The present study aimed to quantify the load-sharing occurring across the ventral spinal structures and the posterior instrumentation, as affected by simple bilateral fixation with and without cages and additional supplementary rods. To achieve this, conventional in vitro flexibility tests were integrated with DIC strain measurements on the ventral spine and with SG analysis on posterior primary rods.

Flexibility tests demonstrated that a posterior bilateral fixation is effective in reducing the RoM and the NZ to negligible values in all motion directions (beyond 98% compared to the intact), except for axial torsion, where a residual instability remained. These trends, consistent for all specimens, were independent on the type of instrumentation considered, in agreement with previous in vitro data on a larger sample size^11–15^. Compared to other studies on degenerative cases without any osteotomy, where XLIF cages were found to provide superior or comparable primary stability characteristics also as a standalone technique in comparison to other cage design (i.e. ALIF)^37,38^, our results may indicate that, despite the preservation of the anterior longitudinal ligament, additional stabilizing features (i.e. anchoring screws, plates) could help achieving superior primary stability also in challenging PSO cases.

Several studies reported that using multi-rod constructs and/or implantation of cages allows to reduce rod strains, which are directly associated to the risk of rod breakage^11–15,18,19^. It is important to remark that, although a cause-effect relationship between the measured strains and the actual loads is quite reasonable, the strain data herein reported should be read as an indirect measure of load effects both on the ventral spine and on the posterior instrumentation. Our strain analysis on primary rods confirmed that usage of interbody cages and supplementary accessory rods is the best strategy to achieve adequate primary stability and to reduce rod strains early after surgery. This is in line with the biomechanical literature, reporting significant differences with supplemental accessory rods and interbody cages compared to the intact condition both in vitro^11–13^ and in silico^18,19^.

Movements in the sagittal plane induced a common and consistent strain pattern for all treated specimens both in flexion and extension, where the anterior spine undergoes axial compression and traction, respectively, while the posterior instrumentation undergoes tension and compression. A similar qualitative response characterized by lower absolute values was observed in lateral bending: the axial compressive strains observed on the compressed (ipsilateral) side of the ventral spine were associated with compression on the posterior contralateral rod and tension on the ipsilateral one because of coupled loading components introduced by the specimens’ enhanced sagittal profile after PSO^39^. In axial torsion, the ventral strains were relatively high and aligned at roughly 45°, with both primary rods experiencing rather comparable strains. These observations relate well with the hypothesized load-sharing effect, where the posterior fixation shielded the whole ventral spine (treated VB and IVD) from reaching the higher strains observed in the intact condition^28^. This was supported by a general significant reduction of ventral strains following PSO in flexion (IVD: about − 88% vs. Intact; PSO level: − 77%), extension (about − 87% and − 86%), lateral bending (− 83% and − 83%) and torsion (− 79% and − 63%), with only specific trends related to the adopted instrumentation.

The strains were higher on the IVD than on the VB, where the presence of mild/moderate osteophytes produced an expected strain-intensification effect^26^. This may indicate that the ventral aspect of the treated vertebra (PSO level) could be more stable, probably because shielded by the posterior instrumentation or by adequate osteotomy closure, thus increasing the load transferred to the anterior column through the intact adjacent discs. Although the mechanical function of the anterior longitudinal ligament (ALL) may be altered following PSO closure, the relatively higher strains noticed on the IVDs adjacent to PSO level may indicate that some residual load is transferred ventrally to the treated vertebra. These mechanisms, coupled to the residual instability measured in torsion^11–17^ may indicate that micromotions could potentially arise on the osteotomy rims if the loads transmitted to the osteotomized vertebra, “floating” among two intact IVDs and “pulled” by the ALL, are sufficiently high. Such an interpretation, supported by our analysis, seems to explain the high rate of revision due to pseudarthrosis as reported by several clinical studies with simple bilateral instrumentation^2–9,40^.

The implantation of cages adjacent to PSO (“PSO-2 + Cages”) did not remarkably affect the ventral strains nor the posterior primary rod strains compared to simple bilateral stabilization. Anyway, the normalized strain on the IVD of specimens #1 and #2 demonstrated a decreasing trend in flexion and in torsion, accompanied by a parallel slight decrease of primary rods strains. This may be attributed to a promoted anterior load-transfer through the stiff intervertebral cages, thus reducing the load on the remaining ventral aspects of the treated disc. The rather constant strains observed at PSO level may indicate that the ventral portion of the treated VB is already shielded by the posterior instrumentation and/or adequate load transfer through the anterior column. Even if not differentiating between the superficial ventral aspect of the spine and the anterior column, a previous computational study reported an improved anterior axial load transfer using interbody cages^18,19^. Although not significant, these mechanisms may relate to the slightly higher fusion rate reported using interbody spacers, graft or cages implanted immediately above and/or below PSO level^6,40^. Even if primary rod strains were slightly reduced in flexion and extension after cages insertion (− 17%, − 13% for “PSO-2 + Cages” vs. “PSO-2”, respectively), variations were not significant. These results may contribute to explain the marginal positive effect reported in previous clinical literature in preventing rod failure with simple bilateral fixation with cages^6,40^.

The addition of supplementary accessory rods (“PSO-4 + Cages”) significantly decreased primary rods strains compared to simple bilateral fixation (“PSO-2”) both in flexion (− 50%), extension (− 40%) and axial rotation (about − 40%). Significant rod strain reduction (about − 44%) was also found in “PSO-4 + Cages” compared to “PSO-2 + Cages” in torsion. Although we expected that increased stiffness of the posterior instrumentation could have further shielded the ventral spine, this was not observed. This indicates that usage of accessory rods may be more effective than simple bilateral instrumentation with/without adjacent cages in reducing the risk of rod failure, rather than affecting the ventral spine or promoting load transmission through the anterior column.

The current in vitro study is affected by specific limitations. The adopted quasi-static protocol based on unconstrained pure moments up to ± 7.5 Nm is considered reliable for comparative purposes^43^, although it may not closely describe the complex in vivo loading condition of severely unbalanced patients. Indeed, the current approach is considered highly reproducible and suitable to describe the early post-operative time while controlling the boundary conditions.

The relatively low posterior primary rod strains measured with SG technique on the most critical configuration (“PSO-2”) resulted to be in the linear elastic regime, therefore not describing inelastic effects occurring during spinal rods contouring or long-term fatigue failure^41,42^.

Due to time constraints involved in the preparation of each instrumentation step and the time required for flexibility test repetition, SG analysis, optimal DIC data collection, and postprocessing, three specimens were analysed. Given the consistency between the results herein discussed and the previous literature on a higher sample size^11–15^, the present study could be seen as an original extension of the previous study on intact^26,27^ and PSO-treated^11,12^ spine segments, where the detailed information about the strain distribution on the ventral spine, once integrated with SG analysis on posterior primary rods, successfully elucidated the load-sharing mechanism among different constructs for fixation and fusion following PSO procedure. The same approach could be easily adapted to investigate the biomechanics of other spinal disease (i.e. aetiology of degenerative disc disease) or to either more conservative spinal treatments or surgical procedure.

## Conclusion

The present study demonstrated how the ventral strains, specifically affected by the loading condition and the presence of local osteophytes, considerably decreased following PSO and instrumentation, confirming the effectiveness of posterior load-sharing. Supplemental accessory rods considerably reduced the posterior rod strains only with interbody cages, but the ventral strains were unaffected: this indicates that the load transfer across the osteotomy could be promoted, while explaining the higher fusion rate with decreased rod fracture risk reported in clinical literature.

## Supplementary Information

Supplementary Information 1.

Supplementary Information 2.

## Data Availability

All data generated or analysed during this study are included in the published article and the Supplementary Information files.
