# Supplementary material for: Load-sharing biomechanics of lumbar fixation and fusion with pedicle subtraction osteotomy
Source: Sci Rep. 2021 Feb 11;11:3595. doi: 10.1038/s41598-021-83251-8 (PMC7878841; doi:10.1038/s41598-021-83251-8)
Supplement: Supplementary file 1 — Supplementary Information 1. [file 41598_2021_83251_MOESM1_ESM.pdf]

Load-sharing biomechanics of lumbar fixation and fusion with pedicle subtraction osteotomy

Luigi La Barbera [a] (*), Hans-Joachim Wilke [b], Maria Luisa Ruspi [c], Marco Palanca [c], Christian Liebsch [b], Andrea Luca [d], Marco Brayda-Bruno [d], Fabio Galbusera [e], Luca Cristofolini [c]

[a] Laboratory of Biological Structure Mechanics, Department of Chemistry, Materials and Chemical Engineering "G. Natta", Politecnico di Milano, Milan, Italy

[b] Institute of Orthopaedic Research and Biomechanics, Trauma Research Center Ulm, Ulm University, Ulm, Germany

[c] Department of Industrial Engineering, School of Engineering and Architecture, Alma Mater Studiorum – Università di Bologna, Bologna, Italy

[d] Department of Spine Surgery III, IRCCS Istituto Ortopedico Galeazzi, Milan, Italy

[e] IRCCS Istituto Ortopedico Galeazzi, Milan, Italy

(*) *Corresponding author:*

luigi.labarbera@polimi.it

Laboratory of Biological Structure Mechanics

Department of Chemistry, Materials and Chemical Engineering "Giulio Natta"

Politecnico di Milano

Piazza Leonardo da Vinci 32

20133 Milano

Italy

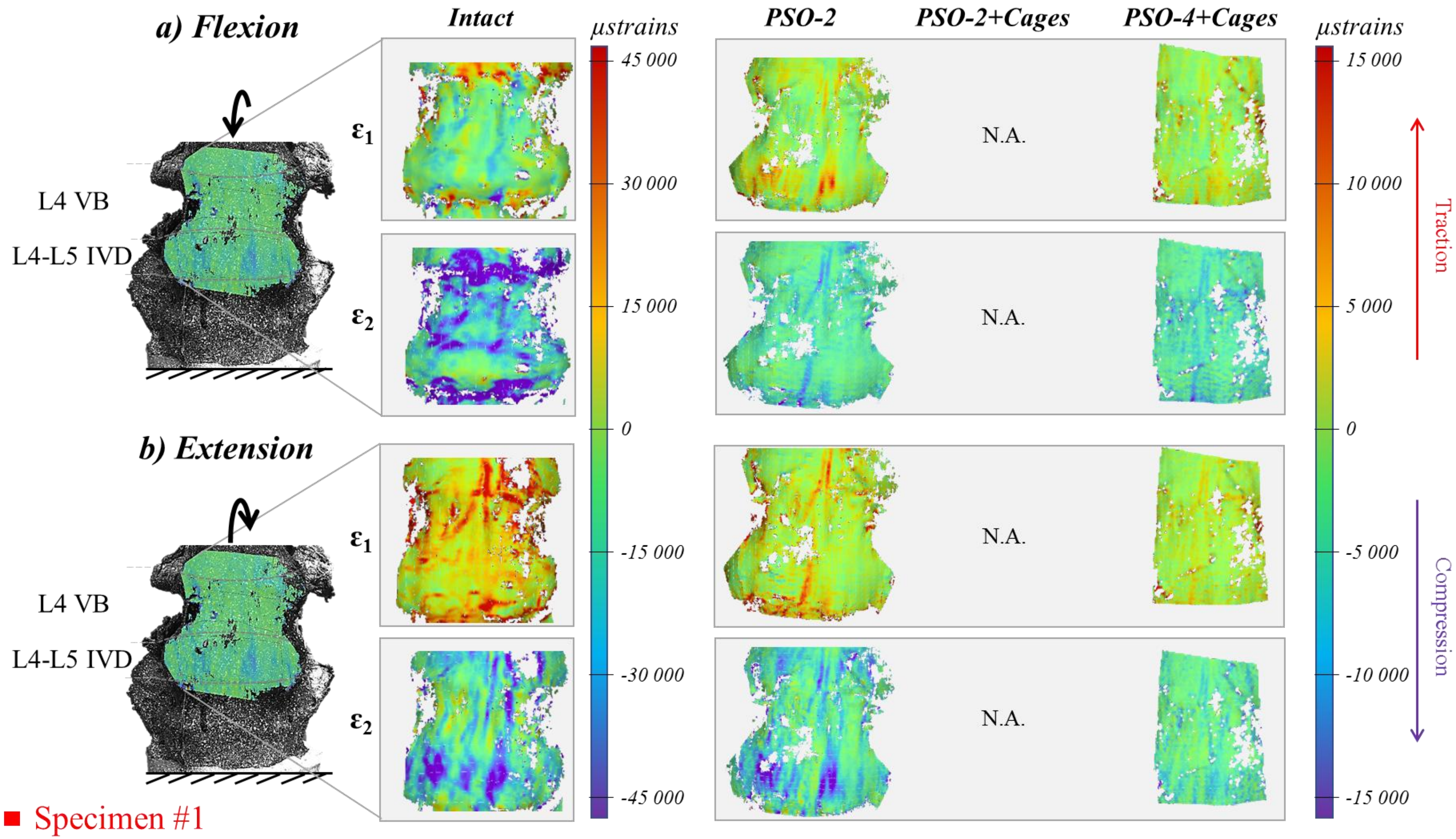

Supplementary Figure 1: Specimen #1 – Flexion (a) and extension (b): tensile (ε_1) and compressive (ε_2) strain maps measured on the "Intact" condition and following PSO at L4 and posterior instrumentation with 2 primary-rods ("PSO-2"), with 2 rods and supplementary intervertebral cages ("PSO-2+Cages"), and with supplementary accessory rods and intervertebral cages ("PSO-4+Cages"). A picture of the specimen with the correlated areas are reported on the left, indicating the treated level (L4) and the caudal IVD (L4-L5). The DIC strain maps have been obtained using the proprietary software Istra 4D (v4.3.1, Dantec Dynamics, Denmark; URL: https://www.dantecdynamics.com/).

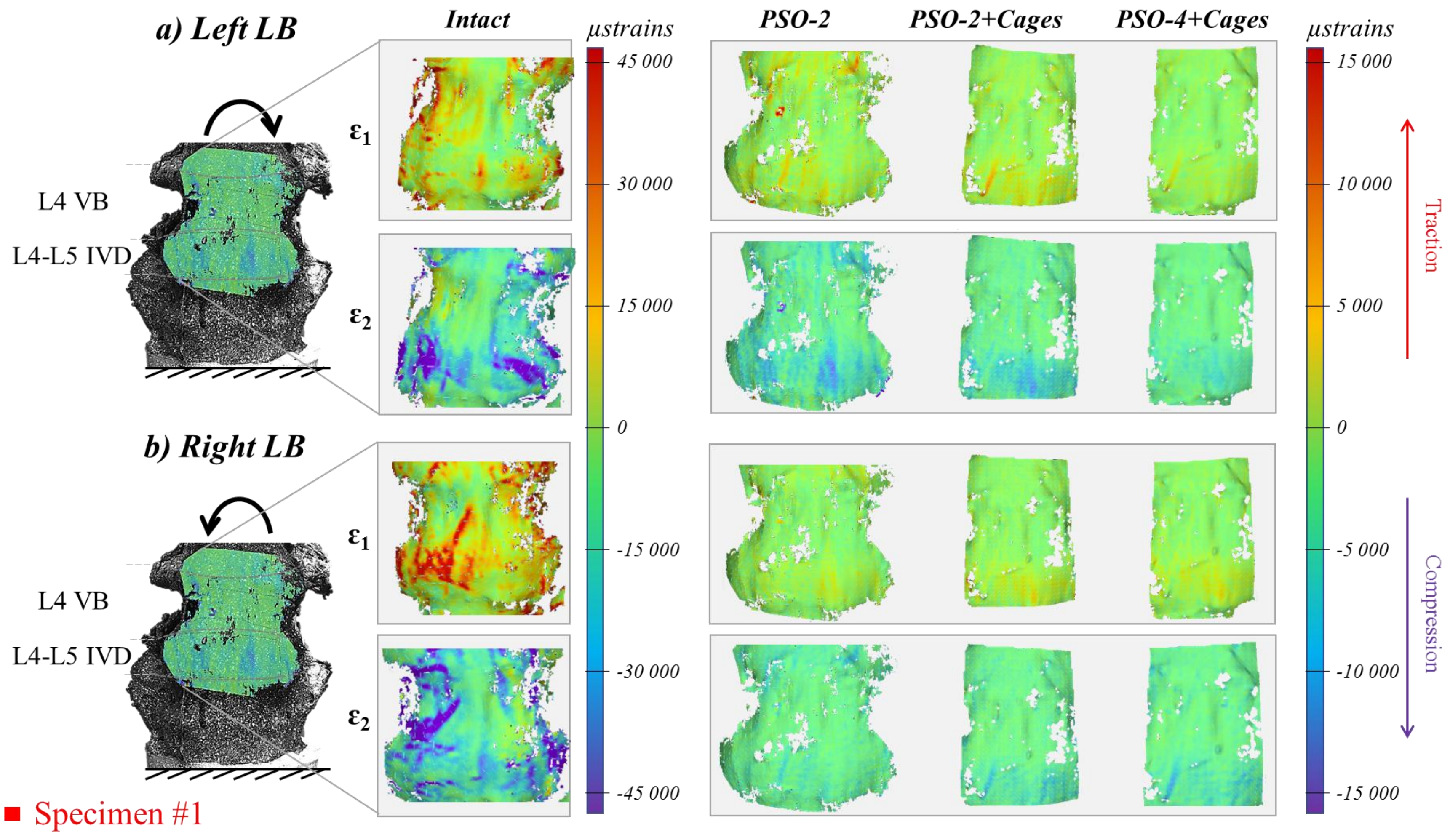

Supplementary Figure 2: Specimen #1 – Lateral bending (LB) left (a) and right (b): tensile (ε_1) and compressive (ε_2) strain maps measured on the "Intact" condition and following PSO at L4 and posterior instrumentation with 2 primary-rods ("PSO-2"), with 2 rods and supplementary intervertebral cages ("PSO-2+Cages"), and with supplementary accessory rods and intervertebral cages ("PSO-4+Cages"). A picture of the specimen with the correlated areas are reported on the left, indicating the treated level (L4) and the caudal IVD (L4-L5). The DIC strain maps have been obtained using the proprietary software Istra 4D (v4.3.1, Dantec Dynamics, Denmark; URL: https://www.dantecdynamics.com/).

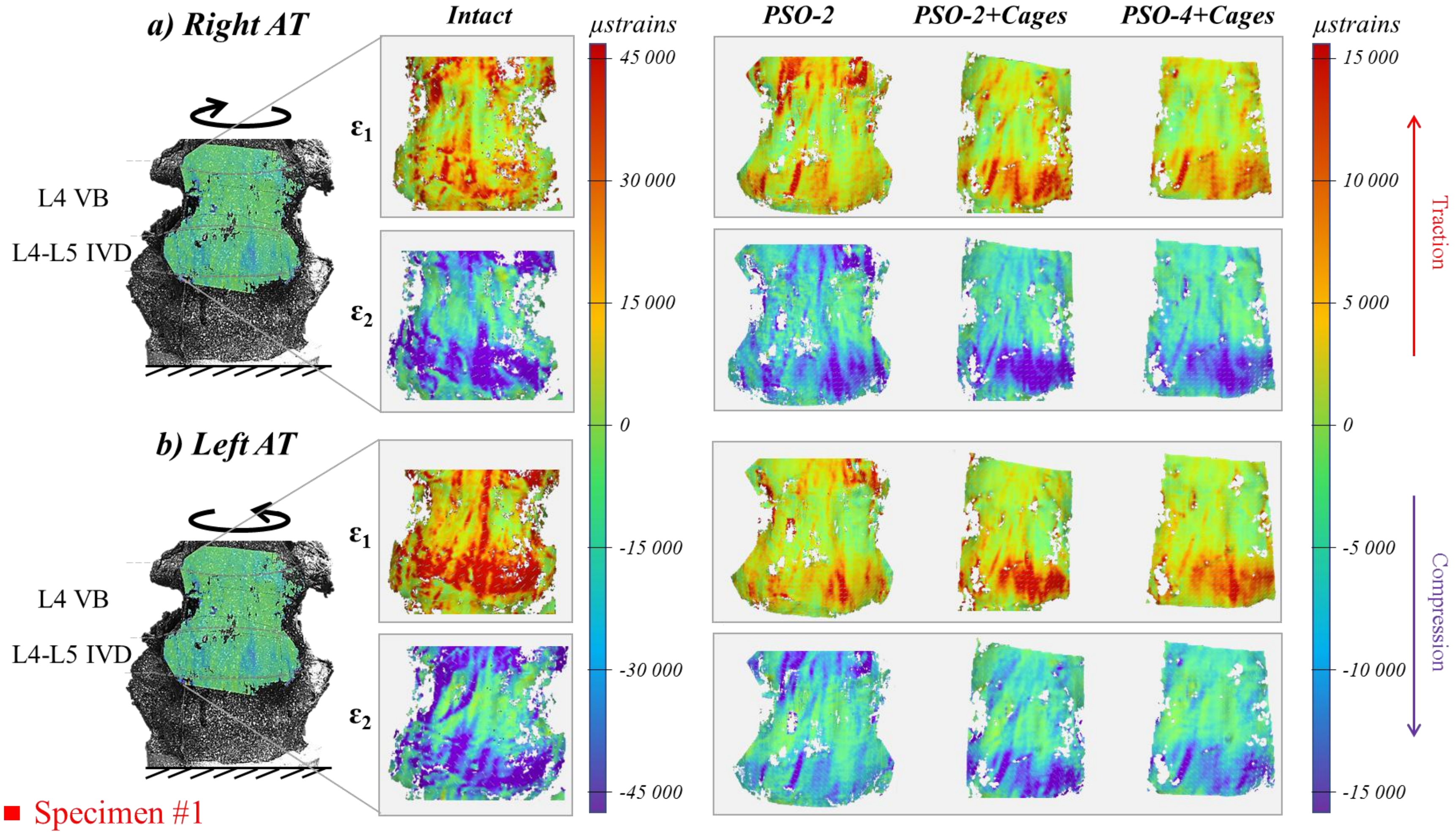

Supplementary Figure 3: Specimen #1 – Axial torsion (AT) right (a) and left (b): tensile (ε_1) and compressive (ε_2) strain maps measured on the "Intact" condition and following PSO at L4 and posterior instrumentation with 2 primary-rods ("PSO-2"), with 2 rods and supplementary intervertebral cages ("PSO-2+Cages"), and with supplementary accessory rods and intervertebral cages ("PSO-4+Cages"). A picture of the specimen with the correlated areas are reported on the left, indicating the treated level (L4) and the caudal IVD (L4-L5). The DIC strain maps have been obtained using the proprietary software Istra 4D (v4.3.1, Dantec Dynamics, Denmark; URL: https://www.dantecdynamics.com/).

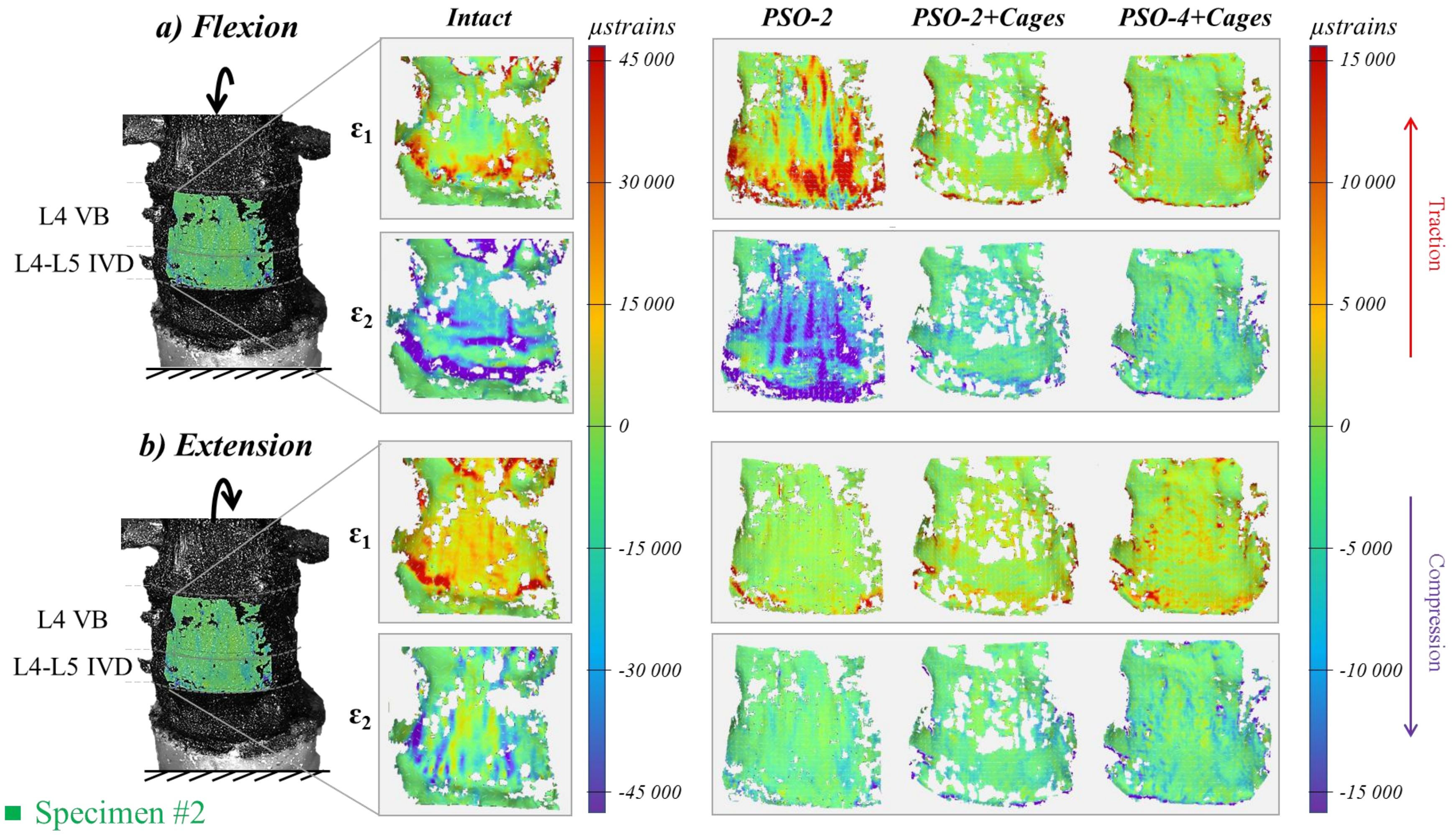

Supplementary Figure 4: Specimen #2 – Flexion (a) and extension (b): tensile (ε_1) and compressive (ε_2) strain maps measured on the "Intact" condition and following PSO at L4 and posterior instrumentation with 2 primary-rods ("PSO-2"), with 2 rods and supplementary intervertebral cages ("PSO-2+Cages"), and with supplementary accessory rods and intervertebral cages ("PSO-4+Cages"). A picture of the specimen with the correlated areas are reported on the left, indicating the treated level (L4) and the caudal IVD (L4-L5). The DIC strain maps have been obtained using the proprietary software Istra 4D (v4.3.1, Dantec Dynamics, Denmark; URL: https://www.dantecdynamics.com/).

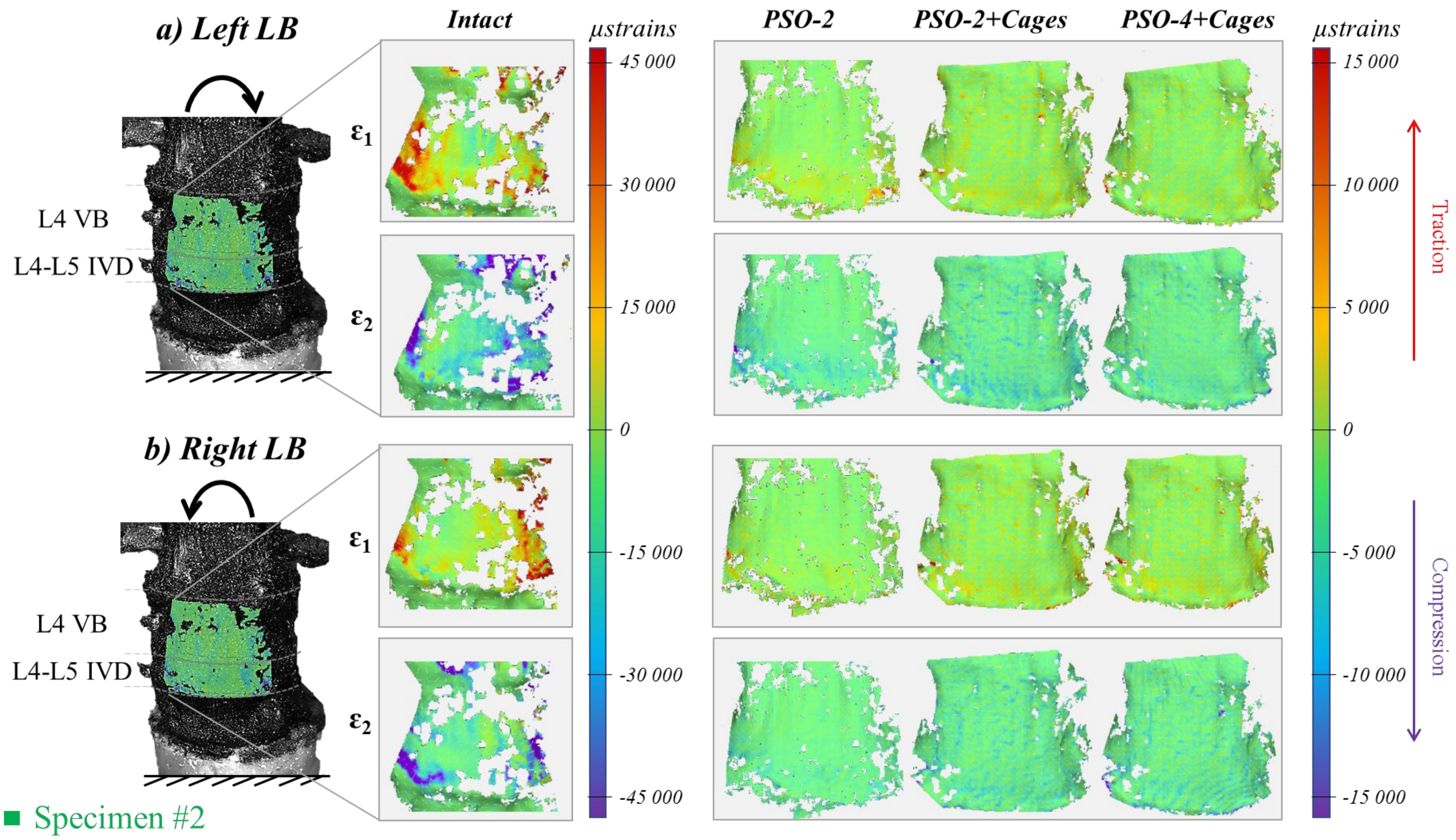

Supplementary Figure 5: Specimen #2 – Lateral bending (LB) left (a) and right (b): tensile (ε_1) and compressive (ε_2) strain maps measured on the "Intact" condition and following PSO at L4 and posterior instrumentation with 2 primary-rods ("PSO-2"), with 2 rods and supplementary intervertebral cages ("PSO-2+Cages"), and with supplementary accessory rods and intervertebral cages ("PSO-4+Cages"). A picture of the specimen with the correlated areas are reported on the left, indicating the treated level (L4) and the caudal IVD (L4-L5). The DIC strain maps have been obtained using the proprietary software Istra 4D (v4.3.1, Dantec Dynamics, Denmark; URL: https://www.dantecdynamics.com/).

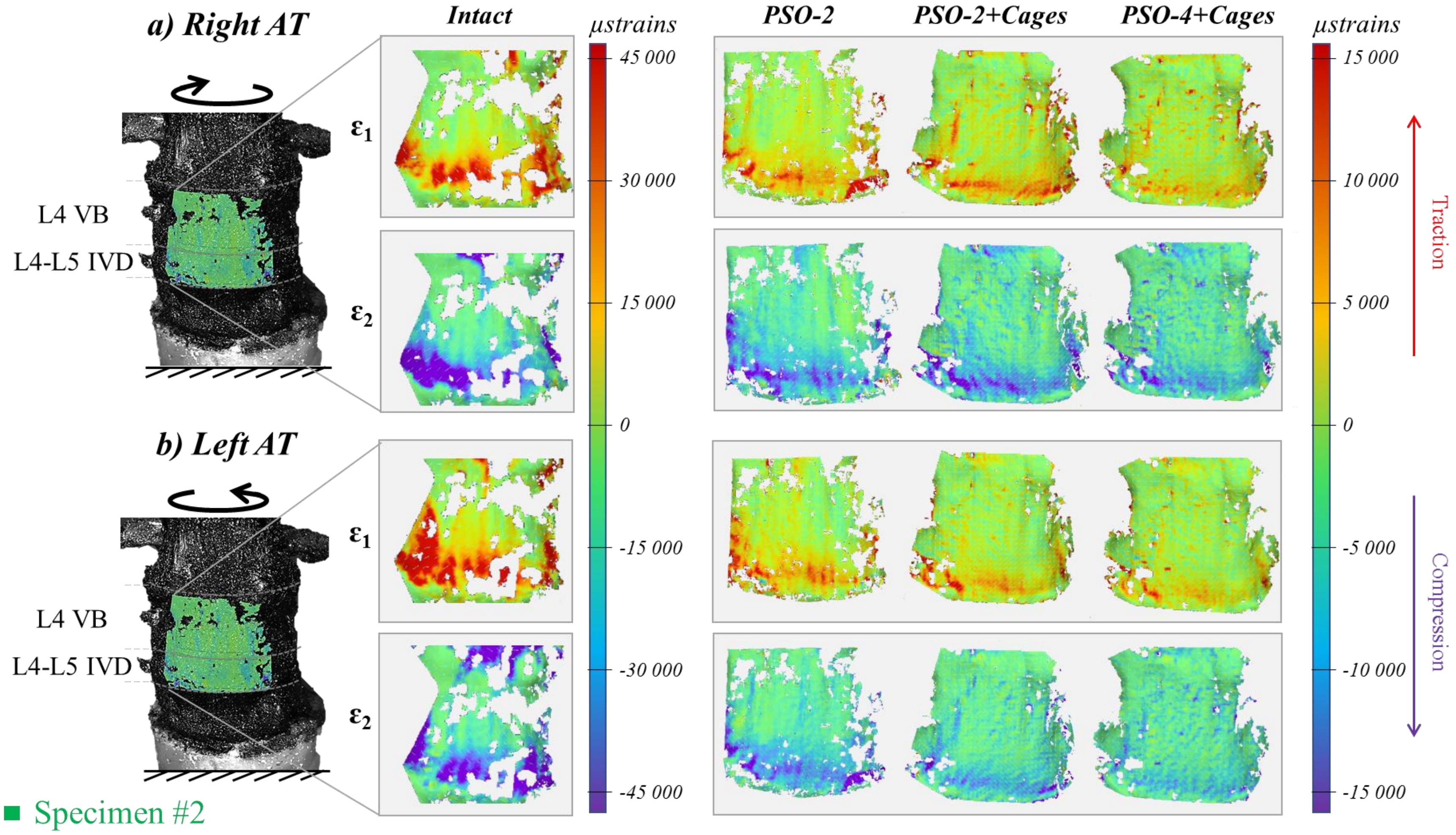

Supplementary Figure 6: Specimen #2 – Axial torsion (AT) right (a) and left (b): tensile (ε_1) and compressive (ε_2) strain maps measured on the "Intact" condition and following PSO at L4 and posterior instrumentation with 2 primary-rods ("PSO-2"), with 2 rods and supplementary intervertebral cages ("PSO-2+Cages"), and with supplementary accessory rods and intervertebral cages ("PSO-4+Cages"). A picture of the specimen with the correlated areas are reported on the left, indicating the treated level (L4) and the caudal IVD (L4-L5). The DIC strain maps have been obtained using the proprietary software Istra 4D (v4.3.1, Dantec Dynamics, Denmark; URL: https://www.dantecdynamics.com/).

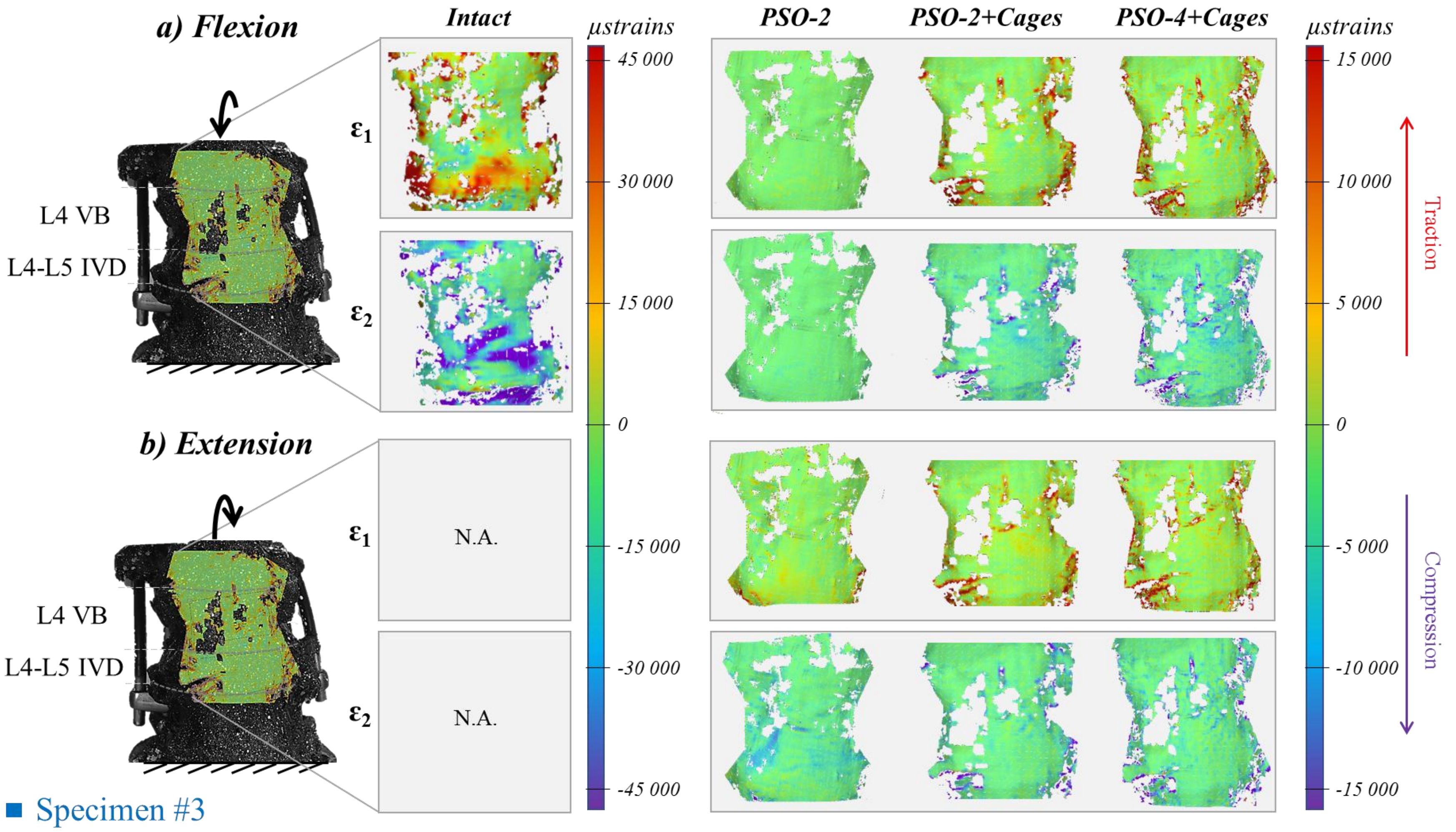

Supplementary Figure 7: Specimen #3 – Flexion (a) and extension (b): tensile (ε_1) and compressive (ε_2) strain maps measured on the "Intact" condition and following PSO at L4 and posterior instrumentation with 2 primary-rods ("PSO-2"), with 2 rods and supplementary intervertebral cages ("PSO-2+Cages"), and with supplementary accessory rods and intervertebral cages ("PSO-4+Cages"). A picture of the specimen with the correlated areas are reported on the left, indicating the treated level (L4) and the caudal IVD (L4-L5). The DIC strain maps have been obtained using the proprietary software Istra 4D (v4.3.1, Dantec Dynamics, Denmark; URL: https://www.dantecdynamics.com/).

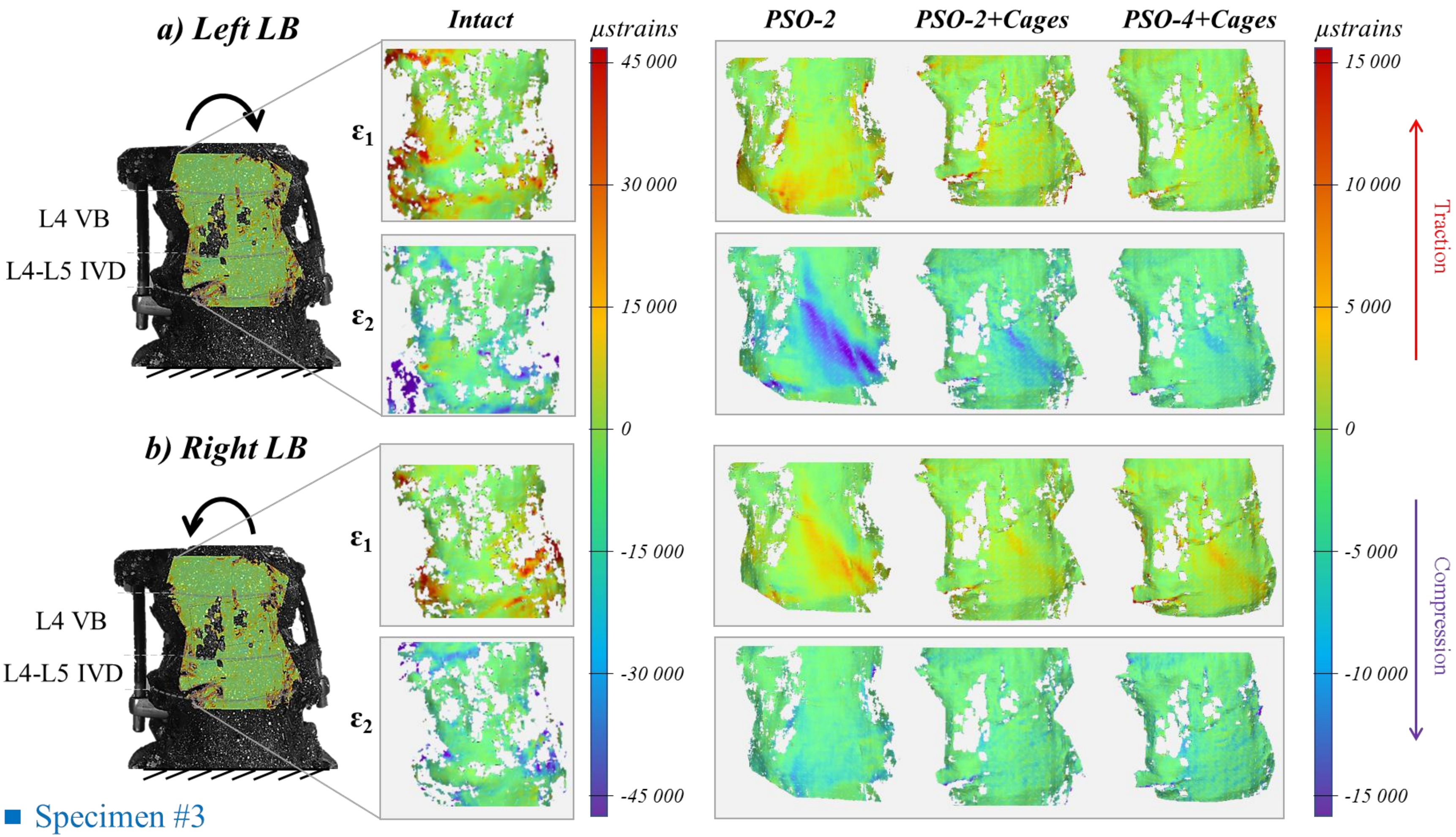

Supplementary Figure 8: Specimen #3 – Lateral bending (LB) left (a) and right (b): tensile (ε_1) and compressive (ε_2) strain maps measured on the "Intact" condition and following PSO at L4 and posterior instrumentation with 2 primary-rods ("PSO-2"), with 2 rods and supplementary intervertebral cages ("PSO-2+Cages"), and with supplementary accessory rods and intervertebral cages ("PSO-4+Cages"). A picture of the specimen with the correlated areas are reported on the left, indicating the treated level (L4) and the caudal IVD (L4-L5). The DIC strain maps have been obtained using the proprietary software Istra 4D (v4.3.1, Dantec Dynamics, Denmark; URL: https://www.dantecdynamics.com/).

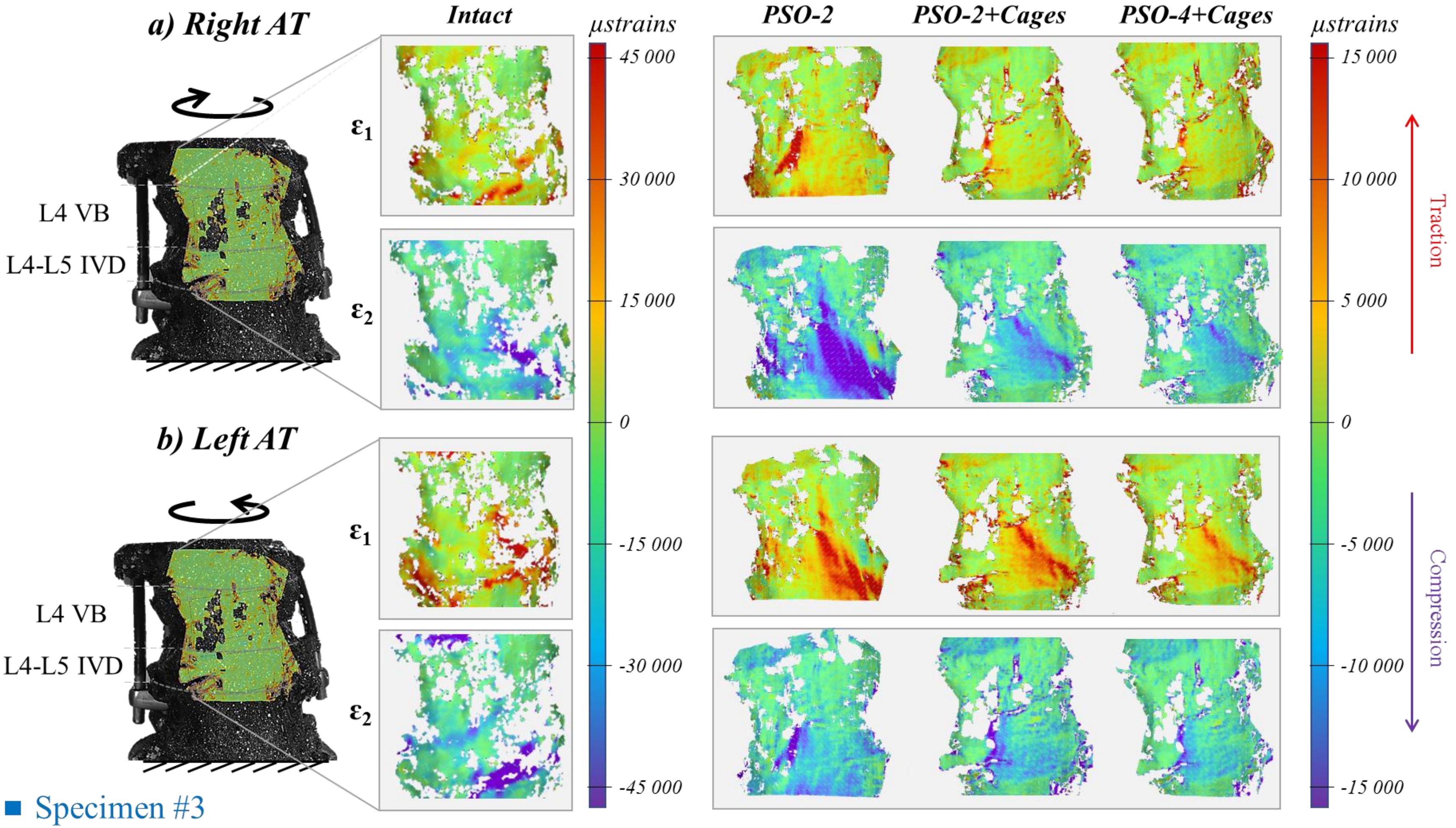

Supplementary Figure 9: Specimen #3 – Axial torsion (AT) right (a) and left (b): tensile (ε_1) and compressive (ε_2) strain maps measured on the "Intact" condition and following PSO at L4 and posterior instrumentation with 2 primary-rods ("PSO-2"), with 2 rods and supplementary intervertebral cages ("PSO-2+Cages"), and with supplementary accessory rods and intervertebral cages ("PSO-4+Cages"). A picture of the specimen with the correlated areas are reported on the left, indicating the treated level (L4) and the caudal IVD (L4-L5). The DIC strain maps have been obtained using the proprietary software Istra 4D (v4.3.1, Dantec Dynamics, Denmark; URL: https://www.dantecdynamics.com/).
