# Supplementary material for: Load-sharing biomechanics of lumbar fixation and fusion with pedicle subtraction osteotomy
Source: Sci Rep. 2021 Feb 11;11:3595. doi: 10.1038/s41598-021-83251-8 (PMC7878841; doi:10.1038/s41598-021-83251-8)
Supplement: Supplementary file 2 — Supplementary Information 2. [file 41598_2021_83251_MOESM2_ESM.pdf]

Load-sharing biomechanics of lumbar fixation and fusion with pedicle subtraction osteotomy

Luigi La Barbera [a] (*), Hans-Joachim Wilke [b], Maria Luisa Ruspi [c], Marco Palanca [c], Christian Liebsch [b], Andrea Luca [d], Marco Brayda-Bruno [d], Fabio Galbusera [e], Luca Cristofolini [c]

[a] Laboratory of Biological Structure Mechanics, Department of Chemistry, Materials and Chemical Engineering "G. Natta", Politecnico di Milano, Milan, Italy

[b] Institute of Orthopaedic Research and Biomechanics, Trauma Research Center Ulm, Ulm University, Ulm, Germany

[c] Department of Industrial Engineering, School of Engineering and Architecture, Alma Mater Studiorum – Università di Bologna, Bologna, Italy

[d] Department of Spine Surgery III, IRCCS Istituto Ortopedico Galeazzi, Milan, Italy

[e] IRCCS Istituto Ortopedico Galeazzi, Milan, Italy

(*) *Corresponding author:*

luigi.labarbera@polimi.it

Laboratory of Biological Structure Mechanics

Department of Chemistry, Materials and Chemical Engineering "Giulio Natta"

Politecnico di Milano

Piazza Leonardo da Vinci 32

20133 Milano

Italy

Supplementary Table 1: Local (L3-L5) range of motion (RoM) and neutral zone (NZ) for each specimen in every loading condition for each instrumentation step.

Specimen	Condition	Flexion/Extension		Lateral Bending		Axial Torsion	
		**RoM (°)**	**NZ (°)**	**RoM (°)**	**NZ (°)**	**RoM (°)**	**NZ (°)**
#1	**Intact**	12.0	3.7	13.6	5.3	8.5	1.8
	**PSO-2**	0.6	0.1	0.0	0.0	1.7	0.1
	**PSO-2+Cages**	0.3	0.1	0.0	0.0	1.5	0.0
	**PSO-4+Cages**	0.2	0.0	0.0	0.0	1.2	0.1
#2	**Intact**	9.7	1.9	12.4	4.4	7.8	0.8
	**PSO-2**	0.5	0.0	-0.1	0.0	1.4	0.1
	**PSO-2+Cages**	0.3	0.1	0.0	0.0	1.5	0.2
	**PSO-4+Cages**	0.4	-0.1	0.0	0.0	1.4	0.2
#3	**Intact**	14.5	5.5	12.8	3.7	5.4	0.8
	**PSO-2**	0.0	-0.1	-0.3	0.0	1.5	0.1
	**PSO-2+Cages**	-0.5	0.1	0.0	0.1	1.5	0.2
	**PSO-4+Cages**	-0.4	-0.1	0.0	0.1	1.4	0.1

Supplementary Table 2: Median [min; max] tensile (ε1) and compressive (ε2) strains measured on the ventral spine, on the sub-ROIs at PSO level (L4 VB) and the IVD below (L4-L5), and on primary rods at full load (±7.5Nm) for each loading condition and instrumentation step.

VENTRAL STRAINS - PSO LEVEL (L4 VB)

Condition		Flexion	Extension	Lateral Bending		Axial Torsion	
				Ipsilateral	Contralateral	Ipsilateral	Contralateral
		Median [min; max] (μstrains)	Median [min; max] (μstrains)	Median [min; max] (μstrains)	Median [min; max] (μstrains)	Median [min; max] (μstrains)	Median [min; max] (μstrains)
**Intact**	ε_1	4419 [3404; 5077] *(*)*	11796 [5595; 17996] *(*)*	5357 [1595; 10642] *(*)*	7076 [2063; 17813] *(*)*	8297 [2187; 18756] *(*)*	6488 [5057; 20052] *(*)*
	ε_2	-15321 [-19389; -15011] *(*)*	-10550 [-19208; -1892] *(*)*	-7679 [-19532; -2055] *(*)*	-3160 [-12002; -39]	-6622 [-10023; -1504] *(*)*	-5066 [-11254; -3328] *(*)*
**PSO-2**	ε_1	1059 [451; 2322]	1524 [450; 3136]	734 [357; 2325] *(§)*	897 [531; 1922] *(#)*	2821 [1647; 4156]	2620 [663; 3686]
	ε_2	-2362 [-6970; -450]	-440 [-1875; -399]	-1074 [-2625; -570] *(§)*	-944 [-1576; -284]	-2554 [-4933; -550]	-2428 [-4761; -1505]
**PSO-2+Cages**	ε_1	1449 [1237; 1660]	2369 [1793; 2945]	1178 [190; 1733]	1136 [780; 7850]	3009 [2001; 5815]	2145 [1438; 10367]
	ε_2	-2692 [-3403; -1980]	-1245 [-1325; -1164]	-939 [-2754; -437] *(§)*	-970 [-1629; -672]	-1935 [-3321; -1603]	-2719 [-4931; -1982]
**PSO-4+Cages**	ε_1	1534 [855; 1963]	2222 [1963; 3108]	910 [378; 1695] *(§)*	915 [575; 1736] *(§)*	2816 [1196; 3916]	1878 [1078; 2177] *(#)*
	ε_2	-2244 [-2591; -2058]	-1479 [-1609; -865]	-959 [-1742; -494] *(§)*	-813 [-1594; -303]	-1742 [-2436; -949] *(§)*	-2256 [-4271; -1586] *(§)*

(*): $p<0.05$ vs. all "PSO" cases.
(#): $p^*<0.017$ vs. "Intact".
(§): $p<0.05$ vs. "Intact".

Supplementary Table 2: …continuation

VENTRAL STRAINS - BELOW PSO LEVEL (L4-L5 IVD)

Condition		Flexion	Extension	Lateral Bending		Axial Torsion	
				Ipsilateral	Contralateral	Ipsilateral	Contralateral
		Median [min; max] (μstrains)	Median [min; max] (μstrains)	Median [min; max] (μstrains)	Median [min; max] (μstrains)	Median [min; max] (μstrains)	Median [min; max] (μstrains)
**Intact**	ε_1	18613 [12948; 19711] *(*)*	20878 [18730; 23025] *(*)*	12048 [2214; 35789] *(*)*	9741 [890; 14448] *(*)*	23552 [5338; 48700] *(*)*	27312 [16590; 50985] *(*)*
	ε_2	-23009 [-23264; -11530] *(*)*	-8319 [-10714; -5923] *(*)*	-11022 [-22819; -4262] *(*)*	-10436 [-27986; -2149] *(*)*	-30291 [-44627; -15277] *(*)*	-22255 [-45939; -7354] *(*)*
**PSO-2**	ε_1	3889 [508; 8800]	1510 [481 ;2365]	2056 [884; 6463]	1450 [1191; 3560]	5245 [3580; 7134]	6362 [2857; 12835] *(§)*
	ε_2	-1392 [-7237; -605]	-1150 [-5014; -565]	-1583 [-3016; -1006] *(#)*	-1796 [-9345; -1237]	-6317 [-14297; -3423]	-5870 [-8309; -4544]
**PSO-2+Cages**	ε_1	1521 [890; 2151]	2275 [1804; 2745]	2502 [1640; 4113]	2097 [1276; 3001]	5556 [2999; 11971]	6337 [3490; 13776] *(§)*
	ε_2	-1954 [-2188; -1720]	-1344 [-1774; -914]	-1949 [-2721; -1380]	-2536 [-4630; -1220]	-5909 [-14516; -4140]	-5946 [-10714; -2039]
**PSO-4+Cages**	ε_1	1334 [1244; 1968]	2071 [1496; 2358]	1558 [1011; 3602] *(§)*	1514 [1003; 1997]	4071 [2944; 8718] *(§)*	5391 [3213; 11280] *(#)*
	ε_2	-1880 [-2227; -1728]	-1713 [-1860; -981]	-1545 [-3539; -1004] *(#)*	-1730 [-2770; -913] *(§)*	-4207 [-6409; -1076] *(#)*	-4681 [-8704; -3061] *(§)*

(*): p<0.05 vs. all "PSO" cases.
(#): p<0.017 vs. "Intact".*
(§): p<0.05 vs. "Intact".

Supplementary Table 2: …continuation

POSTERIOR STRAINS - PRIMARY RODS

Condition		Flexion	Extension	Lateral Bending		Axial Torsion	
				Ipsilateral	Contralateral	Ipsilateral	Contralateral
		Median [min; max] (μstrains)	Median [min; max] (μstrains)	Median [min; max] (μstrains)	Median [min; max] (μstrains)	Median [min; max] (μstrains)	Median [min; max] (μstrains)
**PSO-2**	ε_1	392 [221; 447]	109 [81; 161]	185 [80; 349]	66 [38; 121]	282 [211; 351]	229 [169; 367]
	ε_2	-120 [-139; -74]	-381 [-567; -266]	-78 [-167; -45]	-231 [-402; -130]	-250 [-358; -182]	-283 [-338; -220]
**PSO-2+Cages**	ε_1	291 [178; 383]	96 [70; 137]	175 [81; 325]	64 [34; 122]	296 [236; 344]	237 [149; 362]
	ε_2	-85 [-116; -59]	-329 [-483; -218]	-58 [-157; -36]	-197 [-433;-82]	-238 [-355; -178]	-283 [-346; -210]
**PSO-4+Cages**	ε_1	189 [128; 235] *(**)*	66 [48; 94] *(**)*	155 [86; 267]	53 [26; 79]	188 [101;211] *(**,§§)*	130 [88; 262]
	ε_2	-60 [-68; -43] *(**)*	-232 [-315; -156] *(**)*	-51 [-89; -28]	-172 [-240; -75]	-168 [-313; -101]	-173 [-240; -115] *(**,##)*

(**): p<0.017 vs. "PSO-2".*
(§§): p<0.017 vs. "PSO-2+Cages".*
(##): p<0.05 vs. "PSO-2+Cages".

Supplementary Table 3: Tensile (ε1) and compressive (ε2) strains measured on the ventral spine, on the sub-ROIs at PSO level (L4 VB) and the IVD below (L4-L5), and on primary rods at full load (±7.5Nm) for each specimen in every loading condition and instrumentation step.

VENTRAL STRAINS - PSO LEVEL (L4 VB)

Specimen	Condition		Flexion	Extension	Lateral Bending				Axial Torsion			
					Right		Left		Right		Left	
			(μstrains)	(μstrains)	ipsilateral (μstrains)	contralateral (μstrains)	contralateral (μstrains)	ipsilateral (μstrains)	ipsilateral (μstrains)	contralateral (μstrains)	contralateral (μstrains)	ipsilateral (μstrains)
#1	Intact	ε_1	4419	5595	4347	11351	11975	1945	14965	12233	20052	18756
		ε_2	-19389	-19208	-19532	-2938	-652	-12332	-9103	-9278	-11254	-10023
	PSO-2	ε_1	1059	3136	1664	918	876	575	3350	1874	3686	2470
		ε_2	-2362	-1875	-1384	-761	-1126	-803	-2381	-1936	-3988	-2726
	PSO-2+Cages	ε_1	not available	not available	190	780	1252	808	3760	1990	2300	3318
		ε_2	not available	not available	-827	-773	-672	-437	-1603	-2180	-3258	-2472
	PSO-4+Cages	ε_1	855	2222	388	896	608	378	3057	1078	1655	3115
		ε_2	-2058	-865	-494	-303	-488	-1066	-949	-2334	-2493	-1514
#2	Intact	ε_1	3404	17996	1595	17813	2659	8705	2187	7449	5390	6451
		ε_2	-15321	-1892	-2779	-39	-3935	-12985	-1504	-5419	-3328	-6113
	PSO-2	ε_1	2322	1524	758	726	531	357	1647	663	1570	2217
		ε_2	-6970	-399	-579	-284	-530	-570	-1596	-1840	-1505	-550
	PSO-2+Cages	ε_1	1660	2945	1177	1020	832	1178	2001	1438	3303	2699
		ε_2	-3403	-1325	-794	-1015	-972	-1050	-1949	-1997	-1982	-1797
	PSO-4+Cages	ε_1	1534	3108	918	933	575	916	1196	1466	2101	2089
		ε_2	-2591	-1609	-761	-821	-804	-851	-1717	-2060	-1586	-1766
#3	Intact	ε_1	5077	not available	10642	2800	2063	6367	10144	5527	5057	6400
		ε_2	-15011	not available	-2055	-12002	-3383	-3026	-7130	-4409	-4712	-5214
	PSO-2	ε_1	451	450	2325	1496	1922	709	4156	3366	3523	3171
		ε_2	-450	-440	-2625	-1576	-1179	-1346	-3384	-2920	-4761	-4933
	PSO-2+Cages	ε_1	1237	1793	1733	1533	7850	1271	5815	10367	1988	2651
		ε_2	-1980	-1164	-2754	-1629	-967	-1194	-3321	-4931	-4615	-1921
	PSO-4+Cages	ε_1	1963	1963	903	1736	1735	1695	3916	2120	2177	2575
		ε_2	-2244	-1479	-1742	-1594	-1169	-1707	-1956	-2178	-4271	-2436

Supplementary Table 3: …continuation.

VENTRAL STRAINS - BELOW PSO LEVEL (L4-L5 IVD)

Specimen	Condition		Flexion	Extension	Lateral Bending				Axial Torsion			
					Right		Left		Right		Left	
					ipsilateral	contralateral	contralateral	ipsilateral	ipsilateral	contralateral	contralateral	ipsilateral
			(μstrains)	(μstrains)	(μstrains)	(μstrains)	(μstrains)	(μstrains)	(μstrains)	(μstrains)	(μstrains)	(μstrains)
#1	**Intact**	ε_1	19711	23025	35789	12980	6502	14658	23331	25069	48320	48700
		ε_2	-11530	-10714	-14506	-13797	-27986	-22819	-36727	-45939	-35396	-35254
	**PSO-2**	ε_1	3889	2365	2115	1832	1601	1997	5852	8225	5338	5343
		ε_2	-1392	-5014	-3016	-3539	-1237	-1200	-4934	-4615	-6682	-10263
	**PSO-2+Cages**	ε_1	not available	not available	2783	2885	3001	2221	7305	13776	7079	11971
		ε_2	not available	not available	-2721	-3313	-3608	-1986	-7009	-10714	-7584	-14516
	**PSO-4+Cages**	ε_1	1968	2071	1011	1855	1595	3077	7053	11280	6772	8718
		ε_2	-2227	-1860	-1004	-2491	-1572	-2444	-6409	-8704	-7857	-1076
#2	**Intact**	ε_1	12948	18730	2214	14298	14448	10910	23772	23541	50985	19954
		ε_2	-23264	-5923	-6044	-7074	-2149	-19971	-44627	-17515	-21595	-23263
	**PSO-2**	ε_1	8800	1510	1868	1299	1191	884	7134	2857	6990	4622
		ε_2	-7237	-1150	-2122	-1240	-1255	-1006	-7206	-4544	-8309	-3423
	**PSO-2+Cages**	ε_1	2151	2745	1800	1823	1858	1640	5779	4084	5594	5332
		ε_2	-2188	-1774	-2315	-1759	-1524	-1820	-4432	-4202	-7161	-5581
	**PSO-4+Cages**	ε_1	1334	2358	1832	1003	1997	1231	4340	3213	4009	3782
		ε_2	-1880	-1713	-3539	-913	-1200	-1141	-3627	-3433	-5360	-4174
#3	**Intact**	ε_1	18613	not available	13186	4023	890	4885	5338	16590	29554	31269
		ε_2	-23009	not available	-7538	-16266	-3397	-4262	-15277	-22914	-7354	-25328
	**PSO-2**	ε_1	508	481	4113	3560	1214	6463	3580	12835	5734	5148
		ε_2	-605	-565	-1418	-9345	-2337	-1748	-5428	-5059	-7888	-14297
	**PSO-2+Cages**	ε_1	890	1804	2203	2335	1276	2835	2999	8596	3490	3838
		ε_2	-1720	-914	-1911	-4630	-1220	-1380	-4140	-4730	-2039	-6238
	**PSO-4+Cages**	ε_1	1244	1496	1284	1148	1433	3602	2944	7848	3267	3802
		ε_2	-1728	-981	-1321	-2770	-1888	-1770	-4240	-4002	-3061	-5767

Supplementary Table 3: …continuation.

POSTERIOR STRAINS - PRIMARY RODS

Specimen	Condition		Flexion		Extension		Lateral Bending				Axial Torsion			
							Right		Left		Right		Left	
			right rod (µstrains)	left rod (µstrains)	right rod (µstrains)	left rod (µstrains)	ipsilat. (µstrains)	contralat. (µstrains)	contralat. (µstrains)	ipsilat. (µstrains)	ipsilat. (µstrains)	contralat. (µstrains)	contralat. (µstrains)	ipsilat. (µstrains)
**#1**	**PSO-2**	ε_1	318	221	112	81	228	63	69	139	351	367	245	241
		ε_2	-94	-74	-395	-266	-75	-186	-223	-52	-273	-261	-311	-336
	**PSO-2+Cages**	ε_1	285	178	94	70	205	62	66	146	344	341	246	240
		ε_2	-83	-59	-327	-218	-66	-188	-207	-49	-258	-257	-313	-328
	**PSO-4+Cages**	ε_1	152	128	52	48	184	56	51	126	209	195	144	146
		ε_2	-45	-43	-181	-156	-61	-172	-172	-41	-313	-156	-191	-193
**#2**	**PSO-2**	ε_1	447	394	107	104	80	38	39	141	294	213	210	270
		ε_2	-134	-139	-367	-324	-81	-130	-240	-45	-227	-259	-304	-209
	**PSO-2+Cages**	ε_1	294	289	97	106	81	51	34	122	280	200	229	323
		ε_2	-87	-96	-330	-309	-41	-170	-82	-36	-218	-255	-309	-210
	**PSO-4+Cages**	ε_1	235	170	94	82	86	34	26	112	211	88	117	196
		ε_2	-68	-57	-315	-234	-28	-112	-75	-33	-143	-132	-204	-134
**#3**	**PSO-2**	ε_1	439	389	161	115	327	104	121	349	211	360	169	337
		ε_2	-133	-107	-567	-473	-95	-241	-402	-167	-182	-338	-220	-358
	**PSO-2+Cages**	ε_1	383	316	137	82	287	93	122	325	236	362	149	312
		ε_2	-116	-84	-483	-350	-87	-225	-433	-157	-178	-346	-210	-355
	**PSO-4+Cages**	ε_1	207	226	79	50	267	79	66	213	101	262	95	181
		ε_2	-66	-62	-292	-230	-81	-228	-240	-89	-101	-240	-115	-200
